# *Tm*Relish is required for regulating the antimicrobial responses to *Escherichia coli* and *Staphylococcus aureus* in *Tenebrio molitor*

**DOI:** 10.1038/s41598-020-61157-1

**Published:** 2020-03-06

**Authors:** Maryam Keshavarz, Yong Hun Jo, Bharat Bhusan Patnaik, Ki Beom Park, Hye Jin Ko, Chang Eun Kim, Tariku Tesfaye Edosa, Yong Seok Lee, Yeon Soo Han

**Affiliations:** 1 Department of Applied Biology, Institute of Environmentally Friendly Agriculture (IEFA), College of Agriculture and Life Sciences, Chonnam National University, Gwangju, 61186 Republic of Korea; 2 Department of Biotechnology, Trident Academy of Technology (TAT), F2-A, Chandaka Industrial Estate, Chandrasekharpur, Bhubaneswar, Odisha 751024 India; 3 School of Biotechnology and Life Sciences, College of Natural Sciences, Soonchunhyang University, 22 Soonchunhyangro, Shinchang-myeon, Asan, Chungchungnam-do 31538 South Korea

**Keywords:** Innate immunity, RNAi, Transcription

## Abstract

Relish, a transcription factor, is a critical downstream component of the immune deficiency (Imd) pathway and regulates host defense against bacterial infection by mediating antimicrobial peptide (AMP) synthesis. Understanding the immunological function of the mealworm beetle, *Tenebrio molitor* Relish (*TmRelish*) will be instructive in understanding insect immunity. In the present study, full-length ORF of *TmRelish* was retrieved from *T. molitor-*expressed sequence tags and RNA-seq database. The predicted *Tm*Relish amino acid sequence contained an N-terminal Rel-homology domain; an Ig-like, plexin, and transcription factor domain; ankyrin repeat motifs; a nuclear localization signal; and a C-terminal death domain and shared the highly conserved structure of the Relish proteins of other insect species. *TmRelish* mRNA was detected in all developmental stages of the insect; however, the highest levels were detected in the larval gut tissue and adult hemocytes. *TmRelish* mRNA level was upregulated in the fat body, hemocyte, and gut tissue 9 h after infection of *T. molitor* larvae by the gram-negative bacteria*, Escherichia coli*. Furthermore, *TmRelish* knockdown led to significantly higher mortality of the *E. coli*-infected larvae, and significantly lower mortality of larvae infected with *Staphylococcus aureus* or *Candida albicans*. To elucidate the possible cause of mortality, we measured AMP transcription in the fat body, hemocytes, gut, and Malpighian tubules (MTs) of *T. molitor* larvae. *TmRelish* knockdown suppressed the expression of nine AMP genes in the larval fat body and gut tissue during *E. coli* infection, suggesting that *TmRelish* positively regulates AMP expression in both immune-related tissues, in response to *E. coli* challenge. Furthermore, negative regulation of some AMPs by *TmRelish* in the MTs, gut and hemocytes in response to *C. albicans* infection suggests a crosstalk between the Toll and Imd pathways.

## Introduction

The innate immune response is the first line of defense in vertebrates; however, it is the only form of defense in invertebrates. The lack of a specific adaptive immune response mechanism has conferred the innate immune components of invertebrate species with plasticity in their mechanism of action. Consequently, although similar immune signaling cascades are involved in clearing various microorganisms, invertebrate pathogen surveillance and immune activation systems lack high specificity and memory^1^. Most invertebrates possess a range of cellular and humoral defenses that allow them to overcome infectious agents entering through the gut or exoskeleton/cuticle. In insects, cellular defense mechanisms can be modulated by circulating hemocytes, sessile blood cells that can initiate wound repair/blood coagulation, phagocytosing pathogens in the hemocoel^2^, encapsulating multicellular parasites^3^, and formation of cell aggregates around microorganisms by a process termed nodulation^4^. Humoral insect defense components include antimicrobial peptides (AMPs), prophenoloxidase cascade intermediates, lectins, and complement-like factors. The fat body of insects synthesizes AMPs in response to non-self-microbes. These cationic peptides can disrupt microbial membranes, interfere with bacterial metabolism, and target cytoplasmic components. In *Drosophila*, levels of AMP induction and action have been associated with the type of infectious agent^5^; however, this is not universally true for other insects and invertebrate species. The prophenoloxidase enzymatic cascade causes the release of cytotoxins or opsonins against invading parasites and pathogens, but unlike AMPs, these do not affect host survival following infection^6,7^. Insects also possess numerous lectins that recognize a wide range of pathogens via their carbohydrate moieties and interact with the complement system to promote the lysis of microorganisms^8^.

The humoral immune system of *Drosophila* involves at least three independent signal transduction pathways that lead to the transcriptional induction of AMPs. The Toll signaling pathway is preferentially activated in response to the non-self recognition of fungal and gram-positive bacterial cell surface carbohydrates, such as β-1,3-glucan and Lys-type peptidoglycan, respectively. The Toll pathway requires the Toll ligand spätzle, dorsal, and dorsal group genes such as *Tube, Pelle*, and *Cactus* to activate the transcription of effector AMPs such as drosomycin and metchnikowin (antifungal peptides) and defensin (anti-gram- positive bacterial peptide)^9,10^. In the 18-wheeler-Dif pathway, which is related to the Toll pathway, the Toll-like gene, *18-wheeler*, affects the transcription of antibacterial genes such as *Attacin* instead of diptericin or drosomycin. Both the Toll-dorsal and 18-wheeler-Dif pathways are required for regulation of the AMP, cecropin. The signaling cascade of the 18-wheeler-Dif pathway involves nuclear translocation of dorsal-like immunity factor (Dif) in the absence of Dorsal group genes and the presence of IκB kinase^11^. Conversely, the *meso-*diaminopimelic acid (DAP)-type peptidoglycans found on the cell-surface of gram-negative bacteria are recognized as non-self by the peptidoglycan-recognition proteins (PGRP)-LC and PGRP-LE. Signaling is then modulated downstream by the adapter protein IMD, as part of the IMD signaling pathway, and the NF-κB transcription factor Relish, to induce diptericin expression^12^. NF-κB/Rel proteins, which induces the transcription of robust pro-inflammatory responses with the help of AMPs, therefore appear to be crucial to our understanding of host humoral responses to microbial infection.

The three NF-κB transcription factors, Dorsal, Dif, and Relish share a highly conserved Rel homology domain (RHD; 300 amino acids) and can be grouped into two classes based on their C-terminal RHD sequences. Members of the first class, which includes *Drosophila* Relish (*DmRelish*), are composed of multiple Ankyrin repeats and activate AMP transcription by forming dimers with members of the second class, including Dorsal and Dif. The second class of NF-κB factors contain transactivation domains, which activate AMP gene transcription, although these tend to be less conserved^13^. In *Drosophila*, only Relish, not Dif or Dorsal, is involved in the coordination of the Toll, IMD, and 18-wheeler immunity pathways^14^. Relish activity relies greatly on the uncoupling of its C-terminal IκB-like domain; by the caspase DREDD and IκB-kinase (IKK) complex, which leads to IκB degradation^15^. *DmRelish* homologues have been identified in other insects where they participate in the regulation of AMP genes in response to pathogenic infections. Relish1 and Relish2 from *Anopheles gambiae* and *Aedes aegypti*, respectively, are key transcriptional activators of diptericin/drosomycin (antifungal defense) and defend against gram-positive *S. aureus*^16^. The *DmRelish* homolog of *Culex quinquefasciatus* is activated by the West Nile Virus (WNV), resulting in the triggering of an antiviral response^17^. *Bombyx mori* Relish encodes *Bm*Relish1 and *Bm*Relish2 (dominant negative factor of *Bm*Relish1), which activate or inhibit cecropinB1, respectively^18^. The *Manduca sexta* Relish isoforms *Ms*Rel2A and *Ms*Rel2B have also been shown to induce the transcription of AMP genes^16^. Furthermore, *Relish* silencing in the adult honey bee, *Apis mellifera*, infected with *Escherichia coli* reduced the levels of *abaecin* and *hymenoptaecin* mRNA expression, but not *defensin-1*^19^.

In *Drosophila*, the role of Relish in all three signaling pathways has been explained. Studying the regulatory role of Relish in the IMD signaling pathway has been straightforward; the induction of diptericin and other antibacterial defense genes has been studied by mutational analysis. In the IMD signaling pathway, the endoproteolytic cleavage of Relish by DREDD results in the release of the RHD domain from the C-terminal ankyrin repeat/IκB domain. Subsequently, RHD translocates to the nucleus and initiates the transcription of target genes^20^. Compelling evidence regarding the role of Relish as a master regulator of AMP gene expression following microbial challenge in *Drosophila* and in other insect models has provided important insights into insect humoral immunity. Using the mealworm beetle, *Tenebrio molitor*, we have elucidated intracellular events within the IMD signaling pathway leading to the activation of effector AMPs. We determined the role of *T. molitor* Relish (*Tm*Relish) in regulating the expression of antibacterial and antifungal peptides and the survival of larvae following microbial challenge. We utilized the RNA interference (RNAi) approach to knockdown *TmRelish* in the immune tissues of *T. molitor* larvae and studied the regulation of AMP genes following bacterial and fungal infections.

## Materials and Methods

### Experimental insects and microorganisms

*T. molitor* was reared in an insectary in the dark at 27 ± 1 °C and 60 ± 5% relative humidity (RH). *T. molitor* larvae were fed an artificial diet consisting of 170 g wheat flour, 0.5 g chloramphenicol, 20 g roasted soy flour, 0.5 g sorbic acid, 0.5 mL propionic acid, 10 g soy protein, and 100 g wheat bran in 200 mL of distilled water, autoclaved at 121 °C for 20 min. Only 10^th^–12^th^ instar larvae (approximately 2.4 cm, in length) were used in these experiments.

The gram-negative bacteria *E. coli* (strain K12), gram-positive bacteria *Staphylococcus aureus* (strain RN4220), and the fungus *Candida albicans* (strain AUMC 13529) were used for the immune challenge studies. *E. coli* and *S. aureus* were grown overnight at 37 °C in Luria-Bertani (LB) broth. *C. albicans* was cultured overnight at 37 °C in Sabouraud Dextrose broth. The overnight cultures were harvested by centrifugation at 5000 rpm for 10 min and subsequently washed twice with phosphate buffer saline (PBS, pH 7.0). The density of the cultures was measured at OD_600,_ and the cells were resuspended in PBS at concentrations of 1 × 10^6^ (*E. coli* and *S. aureus*) and 5 × 10^4^ (*C. albicans*) cells/µL, and used to study host-pathogen interactions.

### *In silico* identification and sequence characterization of *TmRelish*

To identify *TmRelish*, a local tblastn analysis was performed using the amino acid sequence of *Tribolium castaneum* Relish (*Tc*Relish) (GenBank: EEZ97717.1) as a query against the locally curated *T. molitor* nucleotide database (derived from *T. molitor* RNA sequencing). The *in silico* results were sufficient to derive the full-length ORF of *TmRelish*. The deduced *Tm*Relish amino acid sequence was used for domain analysis prediction using InterProScan 5.0^21^ and blastx^22^. The nuclear localization signal (NLS) was predicted using cNLS Mapper^23^. To estimate the genetic relatedness of *TmRelish*, multiple sequence alignments were performed using ClustalX2.1^24^, and phylogenetic analysis was performed using MEGA 7^25^. The maximum-likelihood (ML) method was used to construct a phylogenetic tree using the JTT matrix-based model^26^ and the Relish protein of the mud crab, *Scylla paramamosain* (*Sp*Relish: AZK36045.1), was used as the outgroup. To determine the confidence of the branches, we conducted a bootstrap analysis with 1,000 replicates. Multiple sequence analysis and phylogenetic analysis were carried out using amino acid sequences of Relish from orthologous insect groups (at least 15 sequences registered with the NCBI).

### Expression analysis of *TmRelish* mRNA in different developmental stages and tissues

To evaluate developmental expression of *TmRelish* mRNA, samples (n = 20 for each stage) were collected from eggs (EG), young larvae (YL; 10^th^–12^th^ instar), late instar larvae (LL; 19^th^–20^th^ instar), pre-pupae (PP), 1–7-day-old pupae (P1–P7), and 1–5-day-old adults. To investigate tissue-specific expression, the fat body, MTs, gut, integument, hemocytes, ovary, and testis, were dissected from healthy larvae and adults and stored in RNA later solution at **−**20 °C until further use. For *TmRelish* mRNA expression analysis, 20 insects were used from which at least six insects were pooled together as one group (total of three groups). Tissue samples were collected from each group such that three samples for each tissue were obtained. Total RNA was extracted from the samples according to the modified LogSpin RNA isolation method with minor modifications^27^. Briefly, the samples were homogenized with a guanidine thiocyanate-based RNA lysis buffer [20 mM EDTA, 20 mM MES buffer, 3 M guanidine thiocyanate, 200 mM sodium chloride, 40 μM phenol red, 0.05% Tween-80, 0.5% acetic acid glacial (pH 5.5), and 1% isoamyl alcohol in 50 mL], incubated for 1 min at room temperature (approximately 25 °C), and centrifuged at 15,000 rpm for 30 s at 4 °C in a silica spin column (Bioneer, Korea, KA-0133-1). The aqueous phase was discarded, and the genomic DNA was digested by incubating the samples with DNase (Promega, USA, M6101) at 37 °C for 15 min. Thereafter, the silica spin column was washed with 450 mL of 3 M sodium acetate buffer, followed by centrifugation at 15,000 rpm at 4 °C for 30 s. Subsequently, 500 mL of 80% ethanol was added to the spin column and centrifuged at 15,000 rpm at 4 °C for 30 s. After drying the spin column for 1 min, total RNA was eluted in 30 µL of distilled water. For cDNA synthesis, 2 μg of total RNA was incubated with an oligo-(dT)_12–18_ primer at 72 °C for 5 min, 42 °C for 1 h, and 94 °C for 5 min in the AccuPower RT PreMix (Bioneer, Korea) solution.

Quantitative reverse-transcription PCR (qRT-PCR) was used to analyze the developmental and tissue distribution of *TmRelish* with gene-specific primers. The cDNA fragments obtained were diluted at a ratio of 1:20 and qRT-PCR was carried out under the following conditions: denaturation of 95 °C for 20 s followed by 45 cycles at 95 °C for 5 s and 60 °C for 20 s. Relative *TmRelish* mRNA expression was normalized to the *T. molitor* ribosomal protein (*TmL27a*), which was used as an internal control and was amplified under the same conditions. The gene-specific and internal control primers were designed using Primer3.0 plus (http://www.bioinformatics.nl/cgi-bin/primer3plus/primer3plus.cgi); sequence information has been provided in Table 1.

**Table 1:** Sequences of the primers used in the study.

Primer	Sequence (5′-3′)
TmRelish_qPCR_Fw TmRelish_qPCR_Rv	5′-AGCGTCAAGTTGGAGCAGAT-3′ 5′-GTCCGGACCTCATCAAGTGT-3′
TmRelish_Temp_Fw TmRelish_Temp_Rv	5′-TGTGGGAAGATTACGGGAAA-3′ 5′-CAAATTGGCCACGATCTCTT-3′
dsTmRelish_Fw dsTmRelish_Rv	5′-TAATACGACTCACTATAGGGT GACGTGCACCATCAATA-3′ 5′-TAATACGACTCACTATAGGGT GCGTGTTTGGCCTTGAT-3′
dsEGFP_Fw dsEGFP_Rv	5′-TAATACGACTCACTATAGGG TACGTAAACGGCCACAAGTTC-3′ 5′-TAATACGACTCACTATAGGG TTGCTCAGGTAGTGTTGTCG-3′
TmTenecin-1_Fw TmTenecin-1_Rv	5′-CAGCTGAAGAAATCGAACAAGG-3′ 5′-CAGACCCTCTTTCCGTTACAGT-3′
TmTenecin-2_Fw TmTenecin-2_Rv	5′-CAGCAAAACGGAGGATGGTC-3′ 5′-CGTTGAAATCGTGATCTTGTCC-3′
TmTenecin-3_Fw TmTenecin-3_Rv	5′-GATTTGCTTGATTCTGGTGGTC-3′ 5′-CTGATGGCCTCCTAAATGTCC-3′
TmTenecin-4_Fw TmTenecin-4_Rv	5′-GGACATTGAAGATCCAGGAAAG-3′ 5′-CGGTGTTCCTTATGTAGAGCTG-3′
TmDefensin-1_Fw TmDefencin-1_Rv	5′-AAATCGAACAAGGCCAACAC-3′ 5′-GCAAATGCAGACCCTCTTTC-3′
TmDefensin-2_Fw TmDefensin-2_Rv	5′-GGGATGCCTCATGAAGATGTAG-3′ 5′-CCAATGCAAACACATTCGTC-3′
TmColeoptericin-1_Fw TmColeoptericin-1_Rv	5′-GGACAGAATGGTGGATGGTC-3′ 5′-CTCCAACATTCCAGGTAGGC-3′
TmColeoptericin-2_Fw TmColeoptericin-2_Rv	5′-GGACGGTTCTGATCTTCTTGAT-3′ 5′-CAGCTGTTTGTTTGTTCTCGTC-3′
TmAttacin-1a_Fw TmAttacin-1a_Rv	5′-GAAACGAAATGGAAGGTGGA-3′ 5′-TGCTTCGGCAGACAATACAG-3′
TmAttacin-1b_Fw TmAttacin-1b_Rv	5′-GAGCTGTGAATGCAGGACAA-3′ 5′-CCCTCTGATGAAACCTCCAA-3′
TmAttacin-2_Fw TmAttacin-2_Rv	5′-AACTGGGATATTCGCACGTC-3′ 5′-CCCTCCGAAATGTCTGTTGT-3′
TmCecropin-2_Fw TmCecropin-2_Rv	5′-TACTAGCAGCGCCAAAACCT-3′ 5′-CTGGAACATTAGGCGGAGAA-3′
TmThaumatin-like protein-1_Fw TmThaumatin-like protein-1_Rv	5′-CTCAAAGGACACGCAGGACT-3′ 5′-ACTTTGAGCTTCTCGGGACA-3′
TmThaumatin-like protein-2_Fw TmThaumatin-like protein-2_Rv	5′-CCGTCTGGCTAGGAGTTCTG-3′ 5′-ACTCCTCCAGCTCCGTTACA-3′
TmL27a_qPCR_Fw TmL27a_qPCR_Rv	5′-TCATCCTGAAGGCAAAGCTCCAGT-3′ 5′-AGGTTGGTTAGGCAGGCACCTTTA-3′

^※^Underlined region indicates T7 promoter sequences.

### *TmRelish* mRNA expression analysis after microbial challenge

qRT-PCR was conducted to examine the *TmRelish* mRNA induction profiles under microbial challenge. Healthy *T. molitor* larvae (10^th^–12^th^ instar) were infected by injecting 1 µL each of *E. coli* (1 × 10^6^ cells/µL), *S. aureus* (1 × 10^6^ cells/µL), and/or *C. albicans* (5 × 10^4^ cells/µL) into separate sets of larvae (n = 20). Tissue collection, total RNA extraction, cDNA synthesis, and qRT-PCR were carried out as described above. Tissue samples (fat body, hemocytes, gut, and Malpighian tubules) were collected from each set of infected larvae and the PBS-injected mock controls 3, 6, 9, 12, and 24 h post-infection. qRT-PCR was performed using on 20 μL reaction mixture with AccuPower® 2X GreenStar qPCR Master Mix (Bioneer, Korea) and specific primers. Relative *TmRelish* gene expression was calculated using the comparative C_T_ method (2^−ΔΔCT^ method)^28^.

### *TmRelish* dsRNA production and RNAi efficiency

For synthesizing *TmRelish* double-stranded RNA (dsRNA), specific primers, containing T7 promoter sequences, were designed using the SnapDragon-long dsRNA design software (https://www.flyrnai.org/cgi-bin/RNAi_find_primers.pl). The primers were designed to amplify the 851 bp PCR product using AccuPower® Pfu PCR PreMix under the following conditions: denaturation at 95 °C for 2 min followed by 30 cycles of denaturation at 95 °C for 20 s, annealing at 56 °C for 30 s, and extension at 72 °C for 5 min. To synthesize ds*TmRelish*, PCR was carried out under the same conditions. The PCR product was purified using the AccuPrep® PCR Purification Kit (Bioneer, Korea), and the purified PCR product was used to synthesize ds*TmRelish* with the EZ^TM^ T7 High Yield *in vitro* Transcription Kit (Enzynomics, Korea), according to the manufacturer’s instructions. The final PCR product (1 μg, diluted 1:10) was mixed with 4 μL of 5X Transcription Buffer, 2 μL of 10X MgCl_2_, 2 μL of 100 mM DTT, 1 μL of RNase Inhibitor (40 U/μL), 1 μL each of 100 mM rATP, 100 mM rGTP, 100 mM rCTP, and 100 mM rUTP, and 1 μL of RNA polymerase. The mixture was incubated at 37 °C for 3 h and 25 °C for 1 h. The synthesized ds*TmRelish* was then gently mixed with one volume of 5 M ammonium acetate, kept on ice for 15 min, and centrifuged at 13,000 rpm at 4 °C for 10 min. The pellet, containing ds*TmRelish* was washed with 70%, 80%, and 99.9% ethanol and left to air dry. Thereafter, the pellet was resuspended in 30 μL of distilled water (Sigma, USA, W4502-1L). To synthesize double-stranded enhanced green fluorescent protein (ds*EGFP*) as a negative control, a 546 bp PCR product of *EGFP*, derived from the EGFP-C1 plasmid, was used as a template.

Subsequently, 1 μL of the synthesized ds*TmRelish* was injected into one set of larvae (n = 30), and 1 µL of ds*EGFP* was injected into another set of the same stage larvae (n = 30). To investigate the role of *TmRelish* in host-pathogen interactions, healthy *T. molitor* larvae were divided into three sets with two groups each (n = 30 in each group or n = 60 in each set). ds*TmRelish* was injected into one group of each set of larvae, whereas ds*EGFP* was injected into the other group, as a negative control. After *TmRelish* knockdown had been confirmed (24 h after dsRNA injection) in one group of *T. molitor* larvae, 1 × 10^6^ cells/μL of *E. coli* and *S. aureus* and 5 × 10^4^ cells/μL of *C. albicans* were injected into each larva, and larval survival was recorded every day for 10 days. The experiment was conducted at least three times independently to confirm the silencing of the transcripts.

### Effect of *TmRelish* knockdown on AMP expression post-microbial challenge

In order to determine whether *TmRelish* knockdown affected AMP regulation, the expression profile of fourteen AMPs including members of the *Tenecin* family (*TmTenecin-1* (*TmTene1*), *TmTene2*, *TmTene3*, and *TmTene*4)^29–32^; *Attacin* family (*TmAttacin-1a* (*TmAtta1a*), *TmAtta1b*, and *TmAtta*2)^33^; *TmDefensin-1* (*TmDef1*); *TmDef2*; *TmColeptericin-1* (*TmCole1*); *TmCole2*; *TmCecropin-2* (*TmCec2*); and thaumatin-like protein (*TmTLP1* and *TmTLP2*)^34,35^ was examined in *TmRelish-*silenced *T. molitor* larvae following microbial challenge. The immune tissues of the insect (fat body, hemocytes, gut, and MTs) were dissected 24 h post microbial challenge. Total RNA was extracted, and cDNA was synthesized as described above. qRT-PCR analysis was conducted using AMP-specific primers (Table 1). ds*EGFP* and PBS were used as the negative and mock controls, respectively.

### Statistical analysis

Healthy 10^th^–12^th^ instar larvae (approximately 2.4 cm in length) were randomly-selected for all experiments. All the developmental and tissue-specific profiling and microbial challenges tests were independently repeated three times (n = 20 per group). The survival experiments were repeated three times, with 30 larvae per group for each experiment. Values were reported as mean ± SE, and data were subjected to one-way analysis of variance (ANOVA) using SAS 9.4 (SAS Institute Inc., New South Wales, Australia). To evaluate the difference between groups (*p* < 0.05), Tukey’s multiple range tests were performed. The results for the survival assay were analyzed using a Kaplan-Meier plot (log-rank chi-square test) in Excel (http://www.real-statistics.com/survival-analysis/kaplan-meier-procedure/real-statistics-kaplan-meier/). Relative AMP gene expression was calculated using the comparative C_T_ method (2^−ΔΔCT^ method)^28^ and significant differences between ds*TmRelish*- and ds*EGFP*-injected groups were compared using Student’s t-test (*p* < 0.05).

## Results

### *In silico* analysis of *TmRelish*

**Figure 1:**
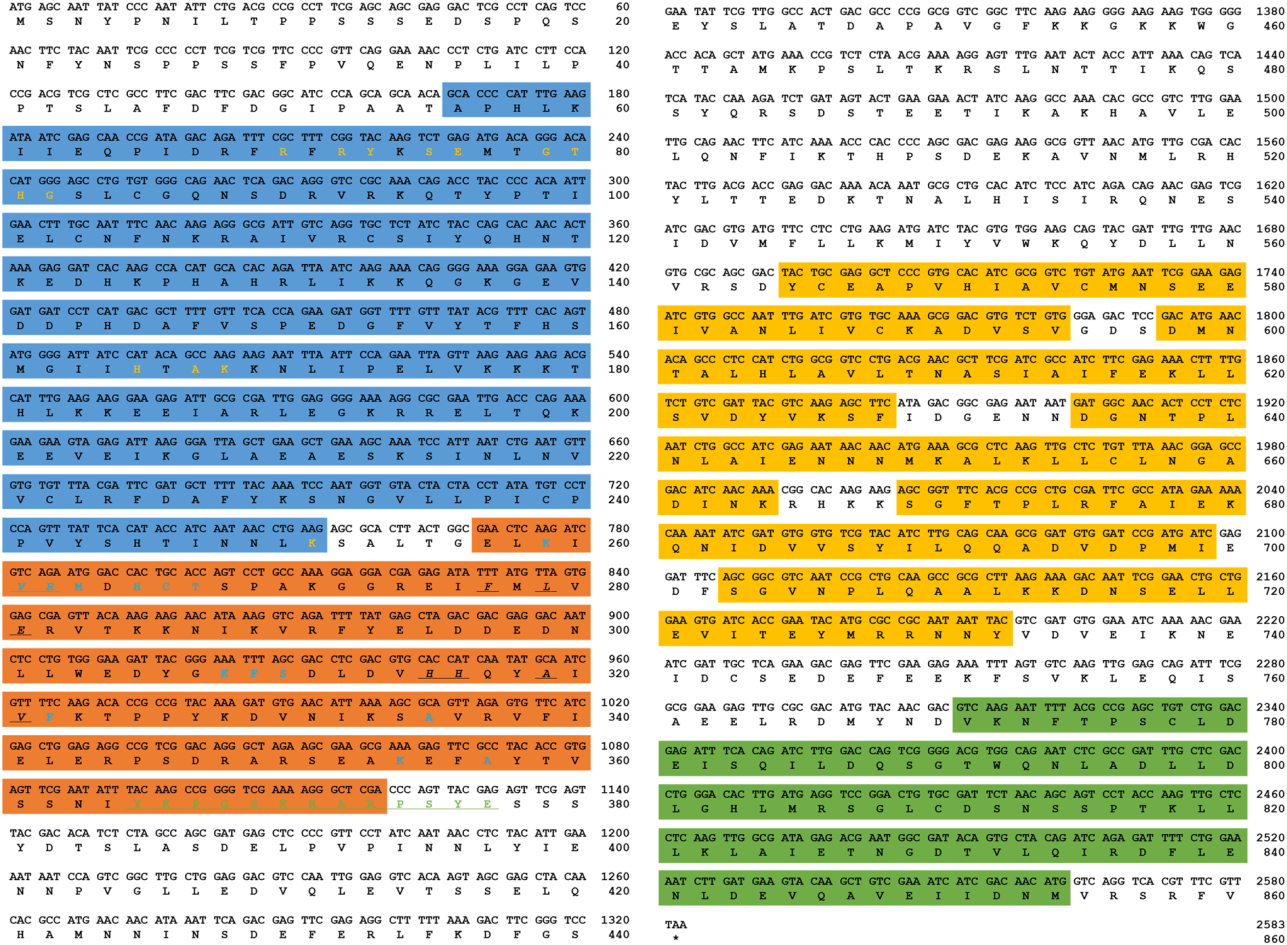
Complete nucleotide and deduced amino acid sequence of *Tenebrio molitor Relish* (*TmRelish)* and its predicted amino acid sequence. Nucleotides and amino acids are numbered along the right margin. The InterProScan program (http://www.ebi.ac.uk/Tools/pfa/iprscan5/) was used to annotate the Rel homology domain (RHD), shown in blue (the DNA binding motif R_68_FRFRYKS_75_ has been underlined), the Ig-like, plexins, transcription factors domain (IPT) in orange, the death domain (DD) in green, and the nuclear localization signal (Y_365_KPGSKRARPSYE_377_) has been underlined in green. The five Ankyrin repeats in the *Tm*Relish sequence have been shown in yellow.

To identify *Relish* in the mealworm beetle *T. molito*r, we screened the RNA-seq data of *T. molitor*, using the *Tc*Relish amino acid sequence as the query (GenBank: EEZ97717.1). Homology search against the RNA-seq database was performed using the local TBLASTN program. We identified a single *Relish* homolog (*TmRelish*) and studied the features of its sequence bioinformatically. The *in silico-*derived *TmRelish* and its deduced amino acid sequence were formatted using the Ultra-Edit program (https://www.ultraedit.com/) and are shown in Fig. 1. The full-length open reading frame (ORF) sequence of *TmRelish*, GenBank accession number MK863367, consists of 2,583 bp, encoding an 860 residue polypeptide. *Tm*Relish contains an N-terminal Rel homology domain (RHD; A_56_–K_251_), an Ig-like, plexins, transcription factors domain (IPT domain; E_257_–R_373_), a death-like domain (DD; V_771_–M_854_), five ankyrin repeats (ANK; Y_565_–V_594_, D_598_–F_628_, D_635_–K_664_, S_669_–I_699_, and S_703_–Y_732_), and a nuclear localization signal (NLS, Y_365_KPGSKRARPSYE_377_). A putative DNA-binding motif (R_68_FRFRYKS_75_) was found at the N-terminus of the RHD domain. The Relish homolog of the Chinese mitten crab, *Eriocheir sinensis* (*EsRelish*), encodes a polypeptide of 1,214 amino acids and contains the typical RHD, an inhibitor kB (IkB)-like domain with six ankyrin repeats, and a DD^36^. However, *Es*Relish has been shown to contain two NLS sequences in contrast to the single NLS observed in *Tm*Relish. In the decapod crustacean, *Exopalaemon carinicauda*, *Relish* consists of 2,141 bp and encodes a polypeptide of 660 amino acids, with an RHD domain and two NLS sequences, similar to *Es*Relish^37^. In the mosquito *Aedes aegypti*, *AaRelish* occurs as three alternatively spliced transcripts encoding separate proteins. The 3.9-kb transcript, which encodes an RHD and IkB-like domain, is the most predominant, showing similarities with *DmRelish*; the second most predominant transcript encodes an RHD and lacks the IkB-like domain; and the least predominant transcript lacks most of the RHD, but contains an intact IkB-like domain. A serine-rich region was identified immediately downstream of the NLS in *Tm*Relish (S_378_–S_380_), similar to that found in *Dm*Relish and unlike *Aa*Relish, while both *Tm*Relish and *Aa*Relish contained the DD. *Tm*Relish*-*associated conserved domains were compared at the amino acid level by multiple sequence alignment using ClustalX 2.1 (S1 Figure A, RHD; Figure B, IPT; Figure C, Ankyrin repeats; Figure D, DD). The DNA-binding motif (RFRFRYKS) showed higher identity compared to the other RHD regions (S1 Figure).

**Figure 2:**
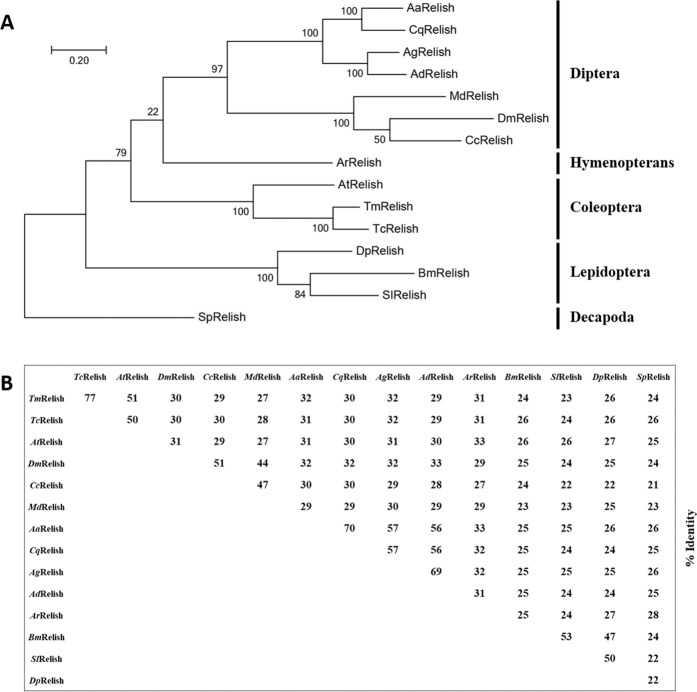
Phylogenetic tree (**A**) and percentage identity analysis (**B**) of the *Tm*Relish and Relish protein sequences of representative insect species. Protein sequences were retrieved from NCBI, as described previously. The phylogenetic tree was constructed using the maximum likelihood method in MEGA7. The percentage of trees in which specific taxa clustered together is given by each branch. Bootstrap analysis values for 1,000 replicates are shown.

The deduced amino acid sequence of *Tm*Relish was compared with Relish sequences from orthologous groups of insects to understand the evolutionary position of *Tm*Relish and to predict functional divergence based on the features of its sequence. Phylogenetic analysis revealed that *Tm*Relish showed close homology with *Tc*Relish and clustered together under the order Coleoptera. Similarly, species belonging to the order Diptera (including the mosquito and *Drosophila*) were clustered together based on their Relish sequences but formed two distinct clades, one for mosquitoes [*Aedes aegypti* (*Aa*Relish), *Culex quinquefasciatus* (*Cq*Relish), *Anopheles gambiae* (*Ag*Relish), and *Anopheles darlingi* (*Ad*Relish)] and one for flies [*Musca domestica Md*Relish), *Drosophila melanogaster* (*Dm*Relish), and *Ceratitis capitata* (*Cc*Relish)]. The phylogenetic tree also revealed sequence divergence within insects and crustaceans, with the decapod *Scylla paramamosain* Relish (*Sp*Relish) formed as an outgroup. Phylogenetic analysis of Relish sequences from *T. molitor* and other representative insects is shown in Fig. 2. Percent identity analysis showed that *Tm*Relish had the highest identity with *Tc*Relish, at 77%, followed by 51% identity with *Aethina tumida* Relish (*At*Relish). Furthermore, *Tm*Relish showed a maximum and minimum identity of 32% (with *Ag*Relish and *Aa*Relish) and 27% (with *Md*Relish), the hymenopteran and dipteran orthologous, respectively, and 23–26% identity with the lepidopteran orthologues.

### Developmental and tissue distribution of *TmRelish*

**Figure 3:**
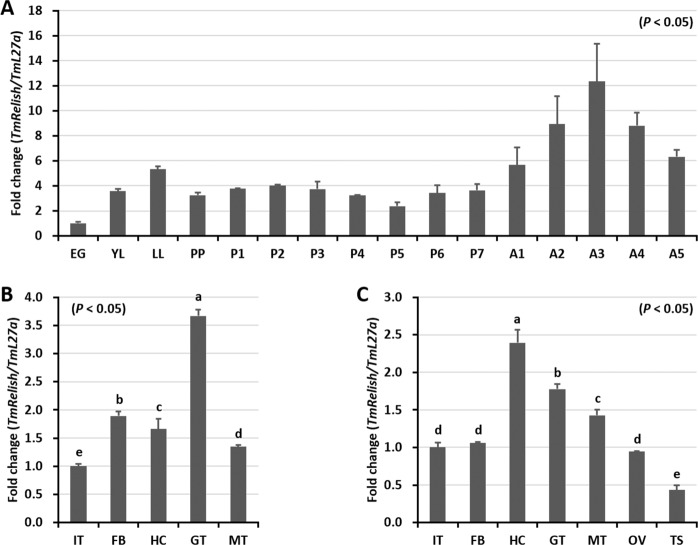
*TmRelish* mRNA expression in developmental stages (**A**) and tissues of *Tenebrio molitor* late-instar larvae (**B**) and 5-d-old adults (**C**) measured by qRT-PCR. (**A**) Relative expression levels of *TmRelish* mRNA in eggs (EG), young larvae (YL), late-instar larvae (LL), pre-pupae (PP), 1–7-day-old pupae (P1–P7), and 1–5-day-old adults (A1–A5) (**B**) Expression of *TmRelish* mRNA in the integument (IT), fat body (FB), hemocytes (HC), gut (GT), and Malpighian tubules (MT) of late-instar *T. molitor* larvae. (**C**) *TmRelish* mRNA expression in the integument (IT), fat body (FB), hemocytes (HC), gut (GT), Malpighian tubules (MT), ovary (OV), and testis (TS) of 5-day-old adults. *T. molitor* 60S ribosomal protein L27a (*TmL27a*) was used as an internal control to normalize RNA levels. Vertical bars represent the mean ± SE.

qRT-PCR was used to detect *TmRelish* mRNA expression in different developmental stages of *T. molitor* (Fig. 3A). *TmRelish* mRNA expression was detected in all developmental stages of the insect, with the highest level of expression observed in the adult stage. The expression of *TmRelish* mRNA in the larval and pupal stages did not change significantly. In the larval tissues examined, expression of *TmRelish* mRNA was highest in the gut, followed by the fat body, hemocytes, and MTs (Fig. 3B). In the 5-day-old adult tissues, *TmRelish* mRNA was expressed at the highest level in the hemocytes, followed by the gut and MTs. Furthermore, the *TmRelish* transcript was weakly detected in the integument, fat body, and ovary. The lowest transcription of *TmRelish* was observed in the testis (Fig. 3C). A higher level of *TmRelish* expression in the gut is expected as Relish acts as the NF-κB transcription factor in the Imd pathway, which is the master regulator of the gut response to microbiota. In the *Drosophila* gut, Relish affects host-microbiota interactions by altering the composition of 16*S* rRNA genes in gut associated microbes^38^.

### Time course analysis of *TmRelish* following microbial challenge

**Figure 4:**
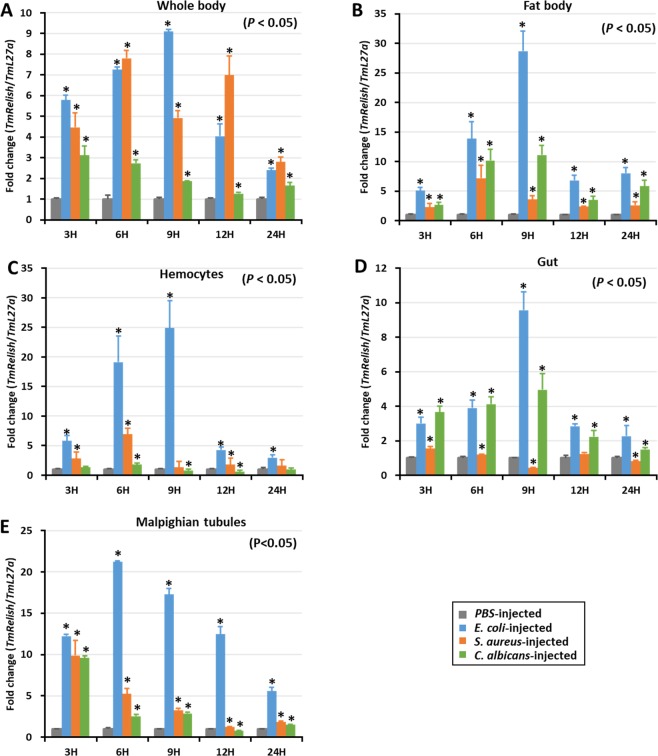
*TmRelish* expression profiles of the whole body (**A**), fat body (**B**), hemocytes (**C**), gut (**D**), and Malpighian tubules (**E**) after *Escherichia coli*, *Staphylococcus aureus*, and *Candida albicans* infection, measured by qRT-PCR. *T. molitor* 60S ribosomal protein *L27a* (*TmL27a*) was used as an internal control. Expression levels of *TmRelish* in PBS-injected mock controls was set to 1. Vertical bars represent the mean ± SE (n = 20). ‘*’ indicates significant difference (*p* < 0.05).

We observed the temporal expression of *TmRelish* mRNA in the fat body, hemocytes, gut, MTs, and the whole-body of *T. molitor* larvae challenged with *E. coli*, *S. aureus*, and *C. albicans* at various time points (3, 6, 9, 12, and 24 h) (Fig. 4). *TmRelish* expression levels were calculated relative to the expression (set to 1) of the mock control (PBS-injected). *TmRelish* mRNA expression was highly upregulated in the whole body of *T. molitor* larvae, challenged with *E. coli*, 9 h post-infection (Fig. 4A). The induction of *TmRelish* expression in the whole body of *C. albicans-*challenged *T. molitor* was also significant (*p* < 0.05), relative to the mock control; however, the expression level was lower than that of the *E. coli* and *S. aureus-*challenged groups. *TmRelish* expression was high 9 h post-infection with *E. coli* in the fat body (Fig. 4B), hemocytes (Fig. 4C), and gut tissue (Fig. 4D)*. TmRelish* mRNA expression was non-significantly different in the hemocytes or gut tissue of *S. aureus*-challenged *T. molitor* larvae; a similar pattern of *TmRelish* expression was recorded in the MTs. Higher level of *TmRelish* mRNA expression was observed in the *E. coli-*challenged group, 6 h post-infection; declining at later time points (Fig. 4E). Hence, the time-course expression data suggests greater induction of *TmRelish* mRNA expression after *E. coli* challenge.

### *TmRelish* gene knockdown and *T. molitor* larval survival

**Figure 5:**
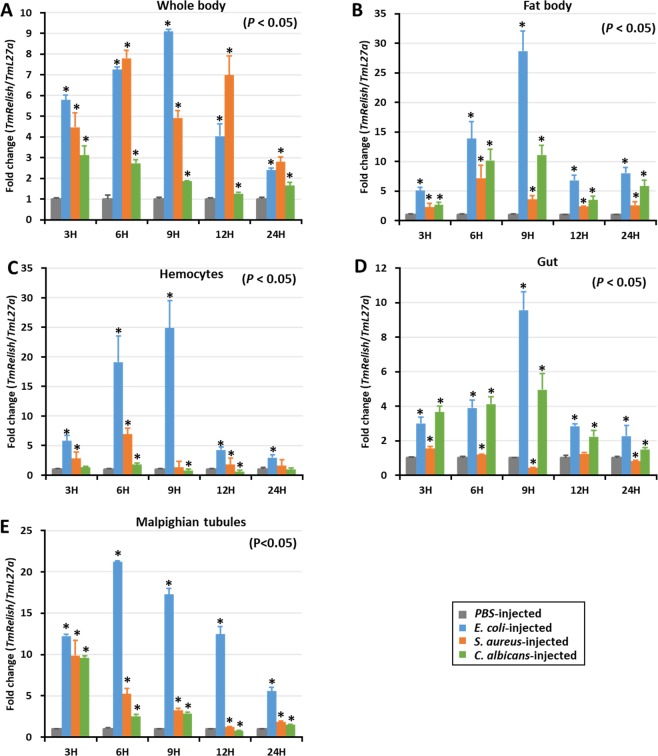
RNAi silencing efficiency of *TmRelish* mRNA in *T. molitor* larvae and survival in the 10 days following microbial challenge. qRT-PCR-based estimation of *TmRelish* knockdown relative to *EGFP* control, 3 days post-dsRNA injection (**A**). Effects of *TmRelish* mRNA knockdown on the survival of *Tenebrio molitor* larvae post *Escherichia coli* (**B**), *Staphylococcus aureus* (**C**), and *Candida albicans* (**D**) infection. ds*EGFP*-treated groups infected with the same microbes were used as negative controls. Survival was monitored for 10 d. The experiment was performed thrice with similar results. ‘*’ indicates significant differences between ds*TmRelish* and ds*EGFP*-treated groups (*p* < 0.05).

Temporal induction of *TmRelish* prompted us to investigate its role in the immune response to microbial challenge. To determine whether *TmRelish* was involved in antibacterial or antifungal defense against *E. coli* and *S. aureus* or *C. albicans*, respectively, we depleted *TmRelish* through RNAi treatment. We found 83% knockdown of *TmRelish* expression upon RNAi treatment, compared to the *EGFP* injected controls on the third day post-dsRNA injection (Fig. 5A). Upon *TmRelish* knockdown, *E. coli*, *S. aureus*, and *C. albicans* were used to challenge *T. molitor* larvae and the survivability was monitored for 10 days. The percent survival of *TmRelish*-silenced larvae significantly dropped (90% mortality) in the *E. coli* challenged individuals (Fig. 5B). Survival of *TmRelish* knockdown larvae also reduced upon *S. aureus* and *C. albicans* challenge to 87% (Fig. 5C) and 80% (Fig. 5D), respectively, when compared to the *EGFP* dsRNA injected control. Taken together, the results suggested the requirement of *TmRelish* for survival of *T. molitor* larvae against *E. coli* infections. *TmRelish* could also be critical in conferring immunity against the gram-positive bacteria *S. aureus* and the fungus *C. albicans*.

### Effect of *TmRelish* knockdown on AMP gene expression

The reduced survival of *TmRelish* knockdown *T. molitor* larvae after being challenged with *E. coli* and other microorganisms suggested a promiscuous role of *TmRelish* in conferring immunity against the pathogens. We hypothesized that *TmRelish* depletion would affect immunocompetent tissues of the insect to produce antimicrobial factors in response to microbial insults. We therefore investigated the expression of fourteen *T. molitor* AMP genes in the *TmRelish* knockdown larval fat body, hemocytes, gut, and MTs post-*E. coli*, -*S. aureus*, and -*C. albicans* challenge.

**Figure 6:**
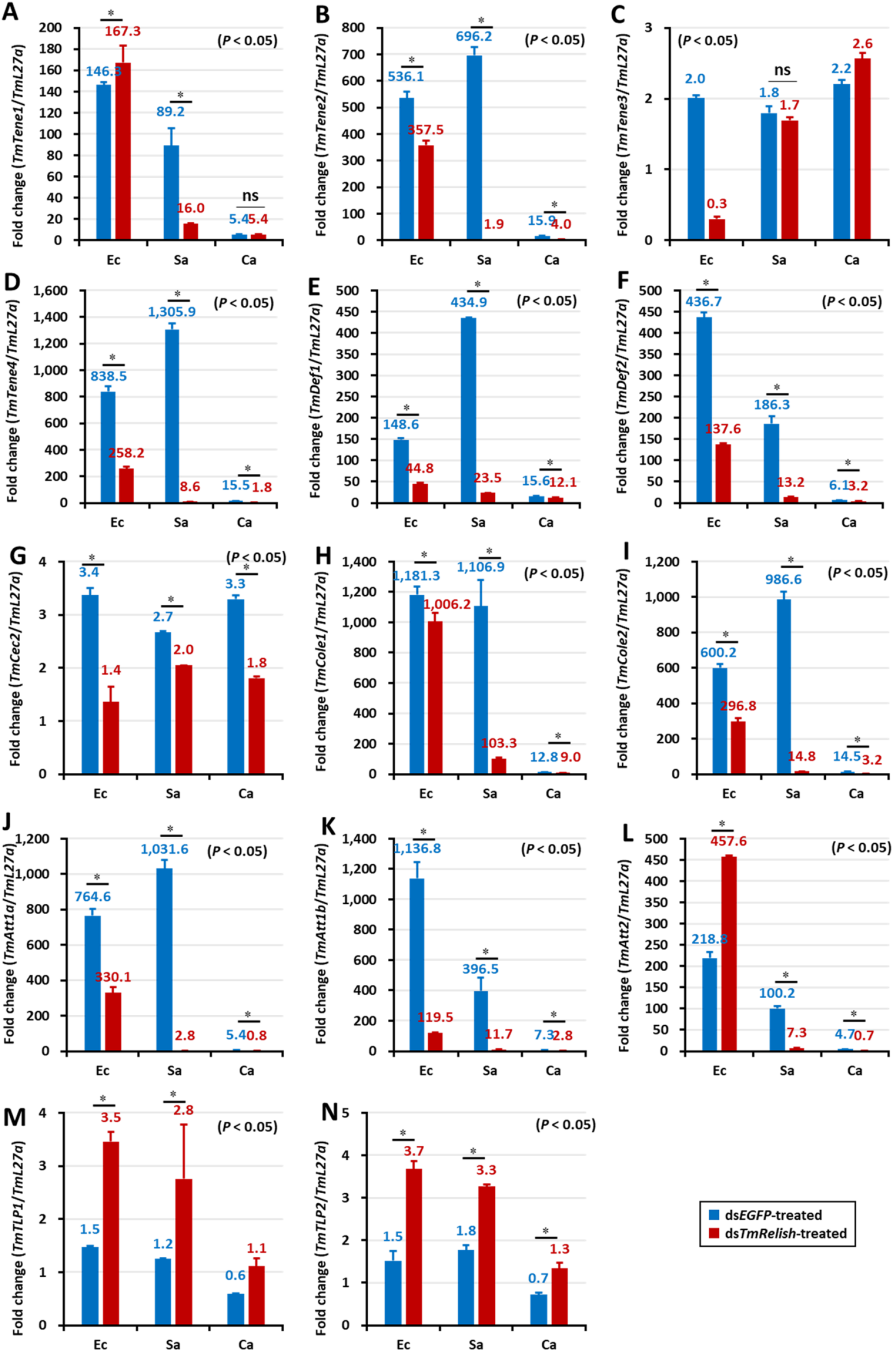
AMP expression levels in the *TmRelish*-knockdown *Tenebrio molitor* larval fat body upon *Escherichia coli* (Ec)*, Staphylococcus aureus* (Sa), and *Candida albicans* (Ca) infection. Healthy larvae (10^th^–12^th^ instar) were injected with ds*TmRelish* and infected with a suspension of *E. coli, S. aureus*, or *C. albicans* on the third day, post-dsRNA injection. PBS-injected larvae were used as controls. After 24 h, the fat body tissue was dissected. Expression level of the AMP genes *TmTenecin-1* (**A**), *TmTenecin-2* (**B**), *TmTenecin-3* (**C**), *TmTenecin-4* (**D**), *TmDefensin1* (**E**), *TmDefensin2* (**F**), *TmCecropin-2* (**G**), *TmColeoptericin-1* (**H**), *TmColeoptericin-2* (**I**), *TmAttacin1a* (**J**), *TmAttacin-1b* (**K**), *TmAttacin-2* (**L**), *TmThaumatin-like protein-1* (**M**), and *TmThaumatin-like protein-2* (**N**) was measured using qRT-PCR and compared with the ds*EGFP*-treated groups. ds*EGFP* was used as a negative control and *TmL27a* as an internal control. The numbers above the bars indicate AMP expression levels. All experiments were repeated thrice, with similar results. Statistical analysis was performed using Student’s t-test (*p* < 0.05); ns: not significant.

In the larval fat body of ds*EGFP*-treated cohorts, the mRNA expression levels of eleven AMP genes showed an increase after microbial challenge (Fig. 6). In *TmRelish* silenced larvae, the mRNA expression of *TmTene2* and -4 (Fig. 6B,D); *TmDef1* and -2 (Fig. 6E,F); *TmCec2* (Fig. 6G); *TmCole1* and *-2* (Fig. 6H,I); and *TmAtta1a* and -*1b* (Fig. 6J,K) was downregulated. Conversely, the expression of *TmTene1* (Fig. 6A) and *TmAtta2* (Fig. 6L) was higher in the ds*TmRelish*-injected group compared to the ds*EGFP*-treated control group, after *E. coli* challenge. However, in *S. aureus* infected *T. molitor* larvae the expression of *TmTene1* and *TmAtta2* was found to be downregulated (Fig. 6A,L). The antimicrobial response of the larval fat body to *S. aureus* infection in ds*EGFP*-treated larvae was strikingly higher compared to the *E. coli* and *C. albicans* infected groups. Notably, AMPs such as *TmTene2* and -4 (Fig. 6B,D), *TmDef1* (Fig. 6E), *TmCole*2 (Fig. 6I), and *TmAtta-1a* (Fig. 6J) were found to show higher expression after *S. aureus* infection. Further, *TmTene3* expression in the fat body of *T. molitor* larvae was not affected in *TmRelish* knockdown individuals, following *S. aureus* or *C. albicans* challenge (Fig. 6C). The antifungal AMPs namely *TmTLP1* (Fig. 6M) and *TmTLP*2 (Fig. 6N) were found to be increased in *TmRelish* knockdown larvae, after microbial infection. The results are in agreement with a previous study on the toll pathway NF-κB factor, *TmDorsal isoform 2* (*TmDorX2*)^39^. Thus, the expression of nine, eleven, and ten AMPs was downregulated in the ds*TmRelish*-injected group after systemic exposure to *E. coli*, *S. aureus*, and *C. albicans*, respectively, suggesting a requirement for *Tm*Relish in regulating AMP gene expression in the fat body.

**Figure 7:**
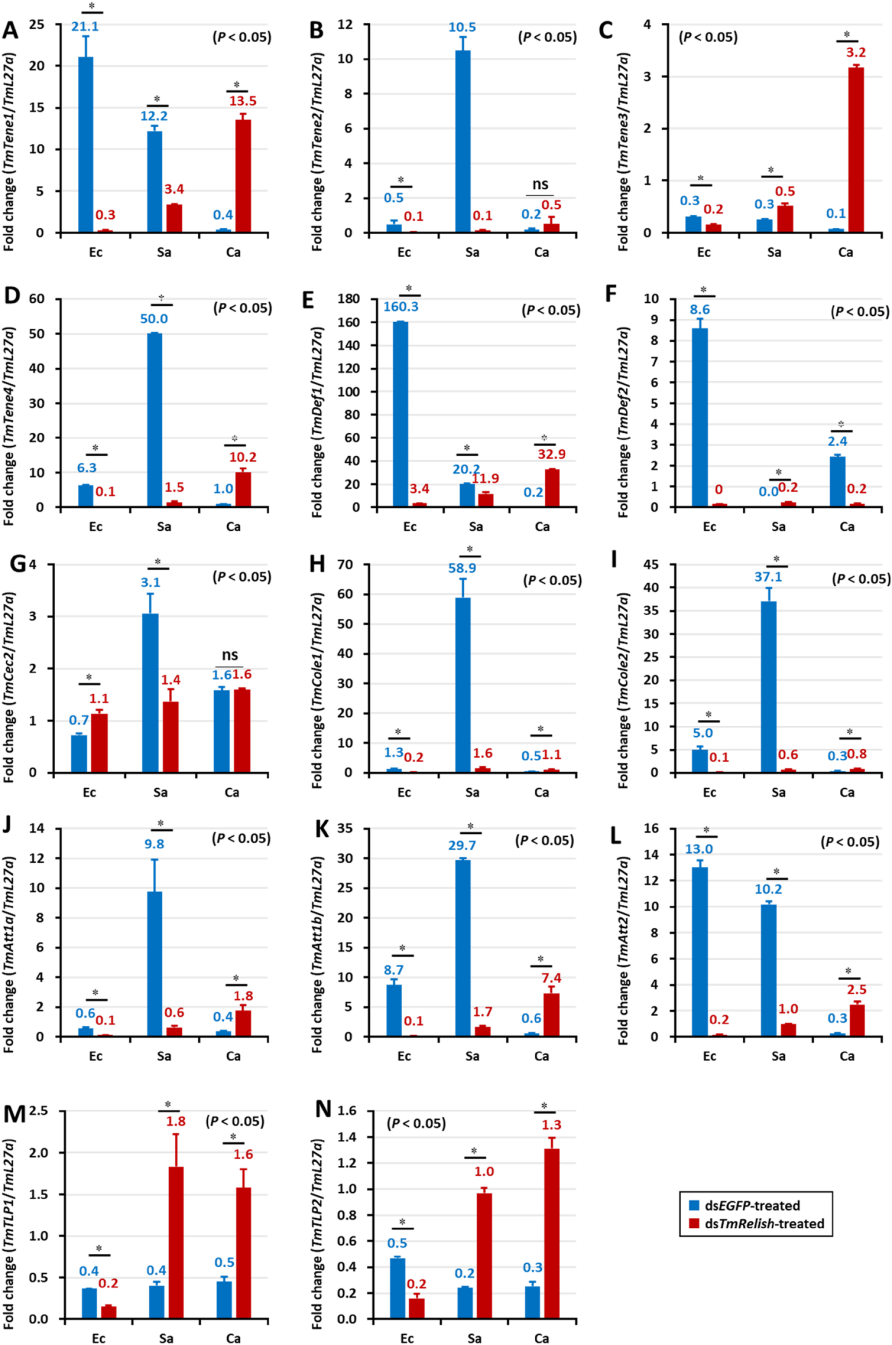
AMP expression levels in *TmRelish-*knockdown *Tenebrio molitor* larval hemocytes upon *Escherichia coli* (Ec)*, Staphylococcus aureus* (Sa), and *Candida albicans* (Ca) infection on the third day post-*TmRelish* silencing. The AMP genes used for analysis include *TmTenecin-1* (**A**), *TmTenecin-2* (**B**), *TmTenecin-3* (**C**), *TmTenecin-4* (**D**), *TmDefensin-1* (**E**), *TmDefensin 2* (**F**), *TmCecropin-2* (**G**), *TmColeoptericin-1* (**H**), *TmColeoptericin-2* (**I**), *TmAttacin-1a* (**J**), *TmAttacin-1b* (**K**), *TmAttacin-2* (**L**), *TmThaumatin-like protein1* (**M**), and *TmThaumatin like protein2* (**N**). ds*EGFP* was used as a negative control and *TmL27a* was used as an internal control. Numbers above the bars indicate AMP mRNA expression levels. All experiments were repeated thrice with similar results. Statistical analysis was performed using Student’s t-tests (*p* < 0.05) and ns: not significant.

Interestingly, the ds*EGFP* group larvae showed lower expression of AMPs in the hemocytes compared to the fat body, gut, and MTs following *E. coli*, *S. aureus*, and *C. albicans* challenge. Moreover, after *E. coli* and *S. aureus* challenge, most of the AMP genes were upregulated in the control groups, while AMP genes were upregulated in response to *C. albicans* in the ds*TmRelish* larvae (Fig. 7). Furthermore, the expression of *TmTene1*, -*2*, and -4 (Fig. 7A,B,D); *TmDef1* (Fig. 7E); *TmCole1* and *-*2 (Fig. 7H,I); and *TmAtta1a*, -*1b*, and -*2* (Fig. 7J–L) was downregulated in the ds*TmRelish-*treated groups in comparison with those in the ds*EGFP*-treated groups, following *E. coli* and *S. aureus* challenges. In contrast, mRNA expression of all AMPs, except for *TmTene2* (Fig. 7B), *TmDef2* (Fig. 7F), and *TmCec2* (Fig. 7G), was upregulated in the *TmRelish* knockdown groups compared to those in the ds*EGFP* group, following *C. albicans* challenge (Fig. 7). These results suggested that *Tm*Relish is not required for eliciting an antimicrobial immune response to *C. albicans* infection^39^.

**Figure 8:**
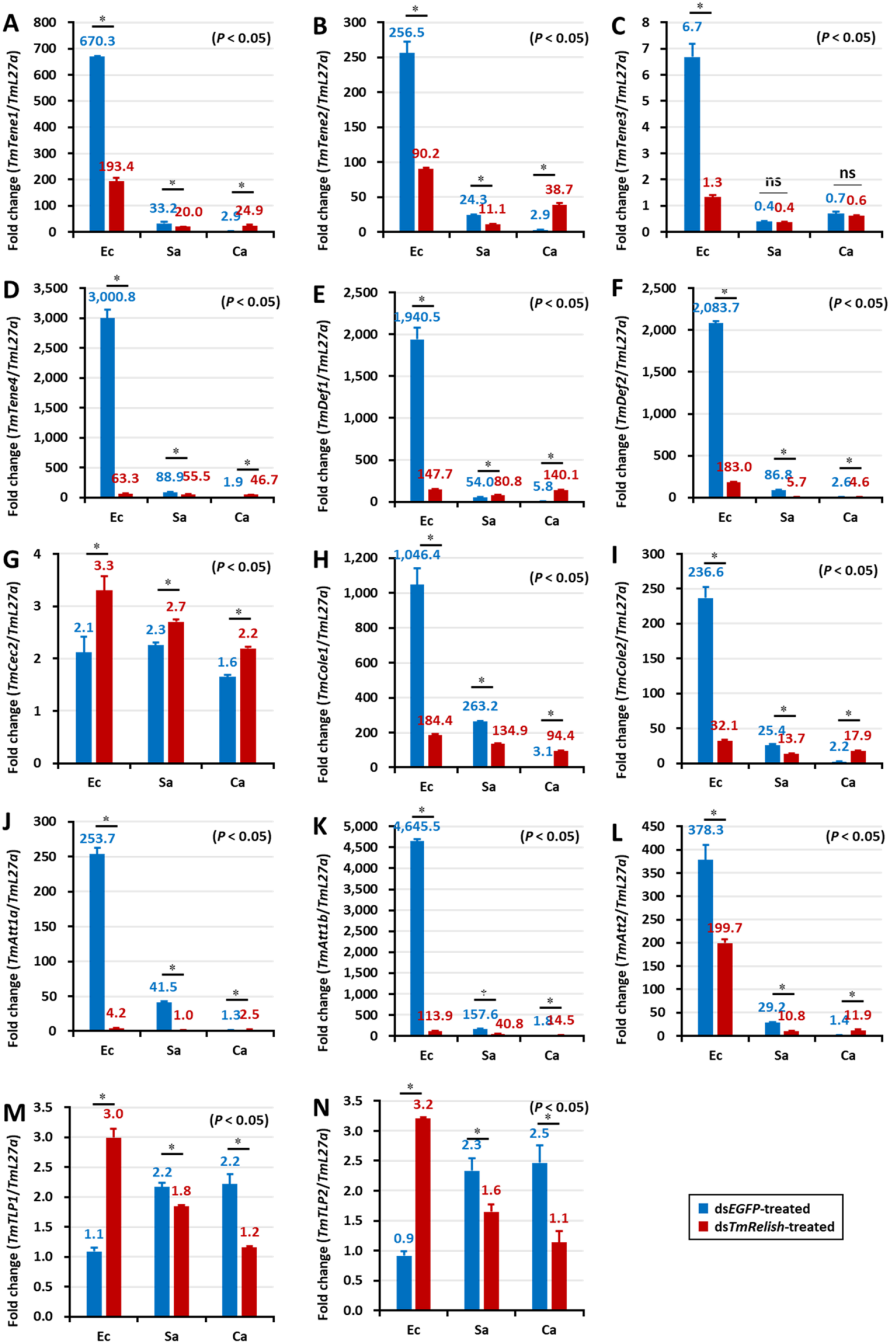
AMP expression level in *TmRelish-*silenced *Tenebrio molitor* gut tissue upon *Escherichia coli*, *Staphylococcus aureus*, and *Candida albicans* infection. qRT-PCR expression profiles of *TmTenecin-1* (**A**), *TmTenecin-2* (**B**), *TmTenecin-3* (**C**), *TmTenecin-4* (**D**), *TmDefensin1* (**E**), *TmDefensin2* (**F**), *TmCecropin2* (**G**), *TmColeoptericin-1* (**H**), *TmColeoptericin-2* (**I**), *TmAttacin-1a* (**J**), *TmAttacin-1b* (**K**), *TmAttacin-2* (**L**), *TmThaumatin like protein-1* (**M**), and *TmThaumatin like protein-2* (**N**). ds*EGFP* was used as the negative control and *TmL27a* was used as an internal control. All experiments were performed at least thrice, and statistical analysis was performed using Student’s t-test (*p* < 0.05); ns: not significant.

**Figure 9:**
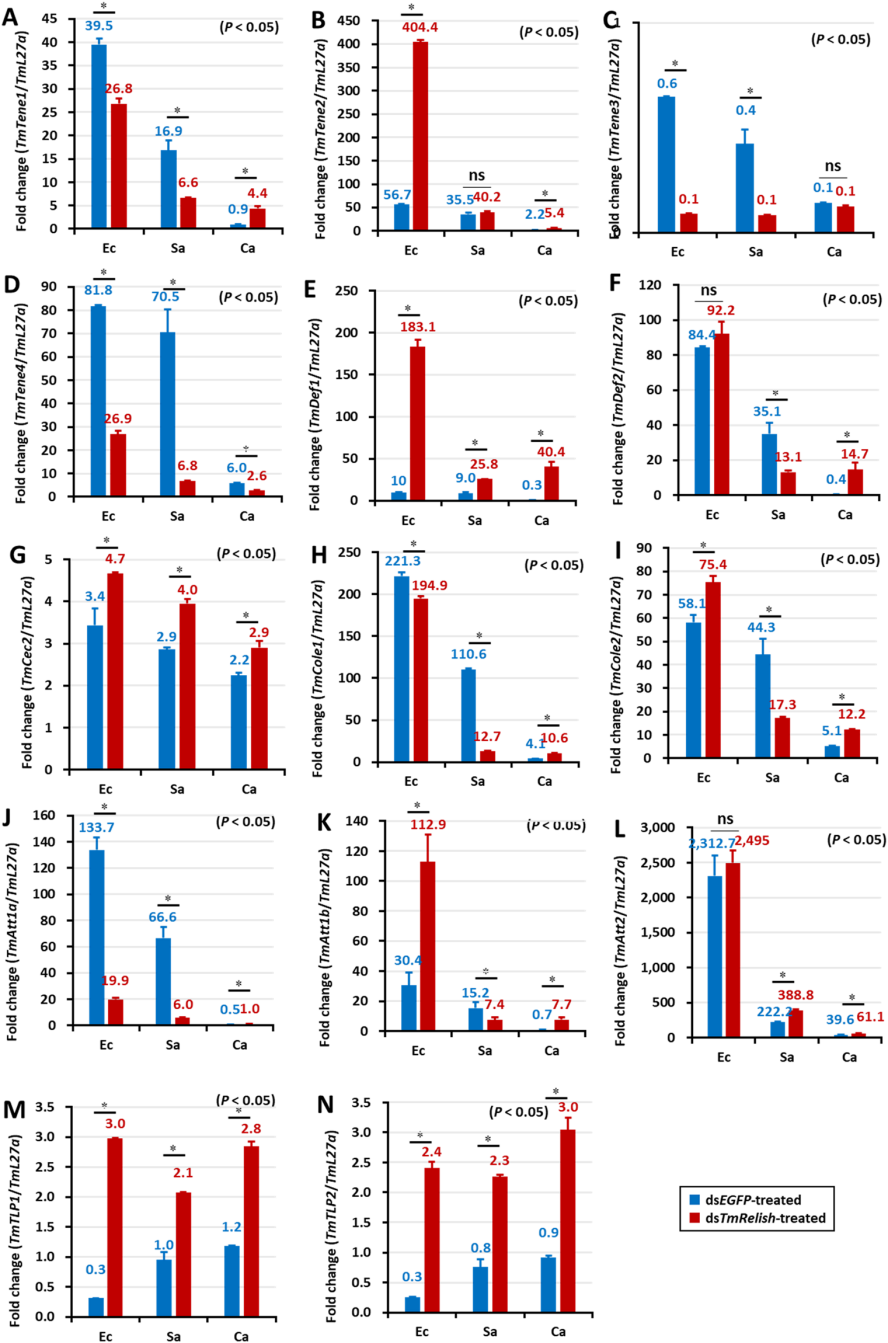
Expression levels of AMPs in *TmRelish-*silenced *Tenebrio molitor* Malpighian tubules upon *Escherichia coli*, *Staphylococcus aureus*, and *Candida albicans* infection. qRT-PCR expression profiles of *TmTenecin-1* (**A**), *TmTenecin-2* (**B**), *TmTenecin-3* (**C**), *TmTenecin-4* (**D**), *TmDefensin-1* (**E**), *TmDefensin-2* (**F**), *TmCecropin-2* (**G**), *TmColeoptericin-1* (**H**), *TmColeoptericin-2* (**I**), *TmAttacin-1a* (**J**), *TmAttacin-1b* (**K**), *TmAttacin-2* (**L**), *TmThaumatin like protein-1* (**M**), and *TmThaumatin like protein-2* (**N**). ds*EGFP* was used as the negative control and *TmL27a* was used as an internal control. All experiments were performed at least thrice, and statistical analysis was performed using Student’s t-tests (*p* < 0.05); ns: not significant.

**Figure 10:**
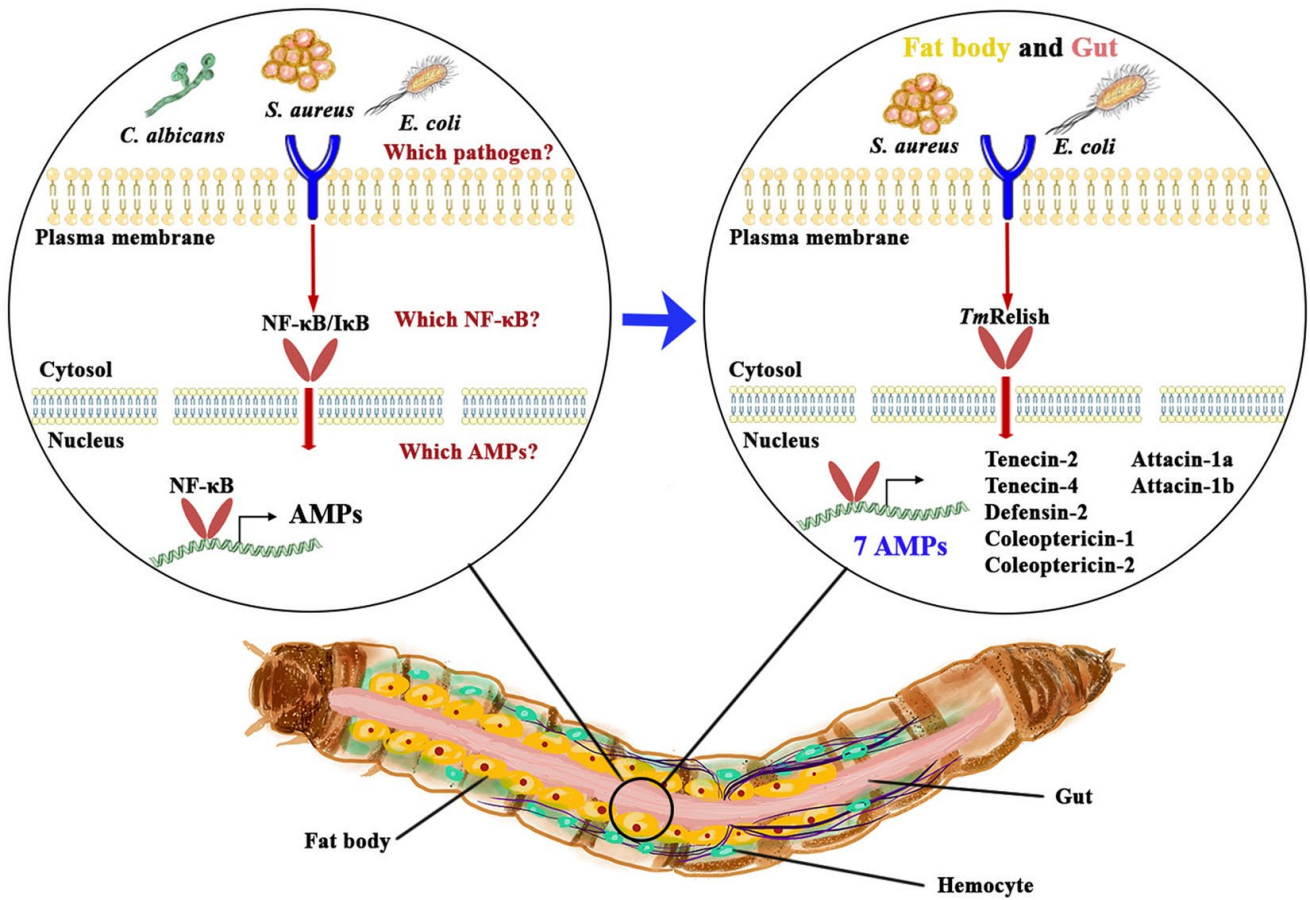
A schematic representation of the humoral immunity pathway positively regulated by Relish in the *T. molitor* fat body and gut, upon *Escherichia coli* and *Staphylococcus aureus* infections, but not *Candida albicans* infection. Seven AMPs were downregulated in the *TmRelish* knockdown group, indicating that Relish is required for survival of the host.

In the gut of ds*EGFP-*treated control larvae, *TmRelish* strongly enhanced *E. coli*-mediated induction of eleven AMP genes including *TmTene1*, -*2*, -3, and -4 (Fig. 8A–D); *TmDef1* and -*2* (Fig. 8E,F); *TmCole1* and -*2* (Fig. 8H,I); and *TmAtta1a*, -*1b*, and -*2* (Fig. 8J–L). This effect was dramatically reduced in *TmRelish* silenced individuals (Fig. 8). In the case of *S. aureus* infection, *TmRelish* was required for the induction of *TmTene1*, -*2*, -*3*, and -4 (Fig. 8A–D); *TmDef1* and -*2* (Fig. 8E,F); *TmCole1* and -*2* (Fig. 8H,I); *TmAtta1a*, -*1b*, and -*2* (Fig. 8J–L), and *TmTLP1* and -*2* (Fig. 8M,N) in the control groups. Further, the AMP genes upregulated after *C. albicans* infection were not affected in *TmRelish* silenced individuals. Notably, eleven AMP genes comprising *TmTene1*, -*2*, -*3*, and -*4* (Fig. 8A–D); *TmDef1* and -*2* (Fig. 8E,F); *TmCec2* (Fig. 8G); *TmCole1* and -*2* (Fig. 8H,I); and *TmAtta1a*, -*1b*, and -*2* (Fig. 8J–L) were upregulated in ds*TmRelish*-injected cohorts. This is relevant, as in an earlier study we have found downregulation of all AMP genes in the ds*TmDorX2*-treated groups, following *C. albicans* exposure. This suggests the importance of *TmDorX2* in the immune response to C. albicans in the gut^39^. In the MTs, following *E. coli* and *S. aureus* infection, five AMPs (*TmTene1*, -*3*, and -*4; TmAtta1a; TmCole1*) were slightly downregulated after ds*TmRelish* injection (Fig. 9A,C,D,J,H, respectively). In addition, *TmCec2* induction was higher in the MTs of *E. coli*, *S. aureus*, and *C. albicans*-challenged *TmRelish* knockdown *T. molitor* larval groups (Fig. 9G). Additionally, knocking down *TmRelish* by RNAi led to reduced expression of eleven, ten, eleven, and seven AMP genes, after *S. aureus* infection, in the fat body, hemocytes, gut, and MTs, respectively. Collectively, our results demonstrate that *TmRelish* promotes *TmTene2*, *TmTene4*, *TmDef2, TmCole1*, *TmCole2*, *TmAtta1a*, and *TmAtta1b* expression in response to *E. coli* and *S. aureus* infections in the larval fat body and gut. We created a scheme summarizing our findings on the functional characterization of *TmRelish* (Fig. 10), swhich shows that *E. coli* infection caused highest mortality in *T. molitor* larvae after the knockdown of *TmRelish*, a component downstream of the Imd pathway, in the fat body and gut. Ten and eleven AMPs were highly upregulated in the fat body and gut tissues following *E. coli* infection, indicating a defense response in the host contributing towards the survival of the larvae. However, upon *TmRelish* knockdown, the expression of these AMPs significantly declined leading to increased host susceptibility to *E. coli* infection.

## Discussion

Beetles constitute 40% of all recognized arthropod species, and their success has been linked to their adaptive plasticity, which enables them to inhabit a variety of environmental niches. This success is unconventionally linked to their robust innate immune system^40,41^. Although genetic studies have elucidated the intricacies of innate immunity in *D. melanogaster*, little is known about the biochemical mechanisms of the innate immune response. The genomes and transcriptomes of beetles such as *T. castaneum*, *T. molitor*, and *Holotrichia diomphalia* have been studied to understand the host defense molecules that regulate immune reactions against a plethora of pathogens.

A genome-wide study of the *T. castaneum* innate immune system revealed information regarding pathogenic and non-pathogenic stress adaptation, and suggested the presence of crosstalk between the immune and stress responses^42^. The study also provided a data resource, which could be used for the discovery and functional characterization of genes involved in innate immunity in other beetle species. *T. molitor* has been shown to attack invading pathogens via transcriptional regulation of AMPs via the toll and Imd signaling cascades^7,32^. In beetles, the Toll pathway is activated by a serine protease cascade, which is activated upon recognition of gram-positive bacteria and fungi, whereas the Imd pathway directly senses pathogens via the peptidoglycan recognition proteins (PRRs) and is controlled by Imd adapter proteins^30,43^. In invertebrates such as beetles, the innate immune signaling components of the Toll pathway are comparatively better understood than those of the Imd pathway. There is, therefore, ample scope for expanding our understanding of the Imd pathway in relation to its modulation of host-pathogen interactions and immune surveillance in the host via effector AMPs.

The present study was conducted to determine the role of the Rel-homology domain (RHD) protein, Relish (downstream of the Imd protein), in *T. molitor* innate immunity and AMP gene regulation. Relish is an important member of the NF-κB transcription factor family and translocates to the nucleus upon the detection of gram-negative bacteria to elicit effector AMP functions. The involvement of Relish in AMP expression has been noted in other invertebrates, including *Drosophila*^36,44^. *Tm*Relish contains RHD, IPT, ANK, and DD domains, and an NLS was identified in the C-terminal portion of the IPT domain. RHD is a characteristic of the eukaryotic Rel protein family and comprises two structural domains: an N-terminal DNA binding domain and a dimerization domain with a C-terminal immunoglobulin-like fold. In addition, *Tm*Relish has an arginine (R)/lysine (K)-rich NLS that mediates the translocation of the protein to the nucleus^45^. Furthermore, *Tm*Relish is a longer form of Relish, as the shorter form contains only the RHD and IPT domains. The short and long forms of Relish are more common in crustaceans such as shrimps; the giant tiger prawn, *Penaeus monodon*, and the giant freshwater prawn, *Macrobrachium rosenbergii*, encode long forms of Relish^36,46^, whereas shorter isoforms have been identified in the Chinese shrimp, *Fenneropenaeus chinensis*, and the white leg shrimp, *Litopenaeus vannamei*^47^. Among insects, both the long and short forms of Relish have been identified in *Anopheles gambiae* and are believed to be formed by the alternative splicing of *Relish2*^48^. In the mosquito, *Aedes aegypti*, both isoforms of *Relish1* were found to be the long forms that activate the Toll-antifungal pathway and induce the expression of AMPs such as diptericin and drosomycin. Furthermore, phylogenetic analysis revealed that *Tm*Relish clustered with the beetle orthologs, supporting their evolutionary position. Interestingly, dipteran Relish separated into two clades: one for mosquitoes and the other for flies. As Relish is essential for mounting an appropriate humoral response against pathogens recognized as non-self via the Imd pathway, it is unlikely that positive selection pressures would have caused the adaptive evolution of the Relish complex, as has been observed in the termite, *Nasutitermes*^49^.

We also examined the expression pattern of *TmRelish* mRNA during development, and observed significantly higher mRNA expression in the adults than in the larvae or pupae. Cross-talk is known to exist between nuclear hormone receptors and innate immunity pathways, suggesting that the juvenile hormone (JH) and 20-hydroxy-ecdysone (20E; steroid hormone) modulate immune responses^50^. Furthermore, JH can act as an immune suppressor whereas 20E can induce AMP expression^51^. The increased *TmRelish* expression observed in adult *T. molitor* can be attributed to low JH levels. Furthermore, we speculate that the increased expression of the extracellular matrix protein, tenebrin, in the late-instar larvae is due to enhanced *TmRelish* mRNA expression. *Tenebrin* mRNA expression was shown to be positively regulated by 20E^52^, suggesting that increased 20E secretion could be related to high *TmRelish* mRNA expression levels in late-instar larvae.

Here, we have reported the biological functions of *TmRelish* in the absence of infection in different tissues. Higher *TmRelish* mRNA expression was observed in immune tissues such as the gut, hemocytes, fat body, and MTs of both *T. molitor* late-instar larvae and 5-day-old adults. In fact, Relish has been shown to be indispensable for eliciting humoral responses via AMP induction in *Drosophila* cuticles and epithelia, including the respiratory and digestive tracts, the MTs, and reproductive organs^53,54^. However, *TmRelish* mRNA expression was not enhanced in the ovary or testis of *T. molitor* adults, in our study. The mRNA of *Relish* in the freshwater prawn, *M. rosenbergii* (*MrRelish*), was also found to be highly expressed in the hemocytes and intestinal tissue^36^. Additionally, a recent study of the tobacco cutworm *Spodoptera litura Relish* (*SlRelish*) has revealed a strong expression in the fat body and hemolymph^55^. Hemocytes and gut compartments are sites of systemic and local inflammatory reactions; thus, the enhanced expression of *TmRelish* in these tissues suggests a role in inflammatory reactions^56^. Upon infection, the Imd pathway in the *Drosophila* gut regulates the shedding of enterocytes into the lumen via Relish, leading to the expression of AMPs to combat the invading pathogenic microorganisms^57,58^. Furthermore, *Drosophila* gut morphology is known to be largely influenced by Imd pathway genes, including the downstream component Relish^38^. In the present study, *TmRelish* mRNA was upregulated in the fat body, hemocytes, gut, and MTs of *T. molitor* late-instar larvae after *E. coli* challenge. The highest level of *TmRelish* mRNA expression was observed 9 h post-infection in the fat body, hemocytes, and gut tissue, and 6 h post-infection in the MTs. Relish is a downstream effector of the Imd signaling cascade, which induces AMP expression in response to microbe-associated molecular patterns (MAMPs), such as *meso-*diaminopimelic acid (DAP)-type peptidoglycan (PGN) which is found in most gram-negative and a few gram-positive (*Bacillus* and *Listeria)* bacteria. Our data demonstrated that *TmRelish* expression was induced in response to *E. coli* and *S. aureus* infection in a manner similar to that of *Anopheles gambiae Relish2*, which responds to both gram-negative and gram-positive bacteria^48^. *TmRelish* mRNA expression levels were lower in the fat body, hemocytes, and gut of the *S. aureus-*infected groups compared with the *E. coli-*infected groups, suggesting that *E. coli* elicit stronger *TmRelish* expression than *S. aureus*. Our results agree with those from an earlier study examining the induction of Relish homologs in the silkworm, *Bombyx mori*, in response to *E. coli* infection^18^. Consistently, *SlRelish* expression was exclusively induced by *E. coli*^55^. The *Relish* transcripts of crustaceans such as the Chinese shrimp, *Fenneropenaeus chinensis*, and the pearl oyster, *Pinctada fucata*, have also been shown to be upregulated in response to infection with the gram-negative bacteria *Vibrio anguillarum* and *Vibrio alginolyticus*, respectively^59^. In conclusion, *TmRelish* is involved in antibacterial immune defense in *T. molitor*.

Loss of certain Relish-dependent target genes, such as AMP genes, has important consequences on humoral immunity. Knocking down the transcriptional activity of *TmRelish* mRNA suppresses expression of AMP genes, considerably weakening the host defense. In this study, survival results showed that when *TmRelish* was silenced, an early, highly significant mortality rate was observed in the *E. coli-*challenged larvae compared to the *S. aureus* and *C. albicans*-infected cohorts. This is consistent with the fact that *Relish* is essential for humoral defense against gram-negative bacteria and that short-term starvation prior to immune challenge increases survival. Furthermore, the knockdown of *A. aegypti Relish2* was shown to dramatically increase the mortality of mosquitoes following infection with gram-positive and -negative bacteria^60,61^. We showed that *E. coli* was capable of killing almost 90% of *TmRelish* knockdown larvae. Thus, *TmRelish* appeared to be essential against *E. coli* infection. Further, *SlRelish*-depleted insects were highly susceptible to *E. coli* insult^55^. Although larval mortality was significant in the *S. aureus-* and *C. albicans*-treated groups, the rate was lower than that in the *E. coli* group.

The production of AMPs is an evolutionarily conserved mechanism, triggered when the cleaved RHD of Relish translocates to the nucleus. AMP expression was shown to be induced in the fat body, hemocytes, and gut tissues of *Drosophila*^54,62^. Analyzing the expression of *T. molitor* AMP genes in larvae with or without *TmRelish* knockdown revealed that expression levels of AMPs decreased upon silencing *TmRelish* during infections; *TmRelish* can therefore be proposed to be a positive regulator of AMPs, and *TmRelish*-silenced larvae are more susceptible to microbial infections compared to controls. Although we observed decreased levels of several AMPs in ds*TmRelish*-treated larvae following *S. aureus*, *TmRelish* depletion led to mild but significant mortality. In a previous study, the mRNA levels of *TmTene1*, *TmTene2*, *TmTene4*, *TmDef2, TmCole1, TmCole2, TmAtta1a, TmAtta1b*, and *TmAtta2* were found to be significantly reduced in *T. molitor* following silencing of immune deficiency (*Tm*IMD), an adapter molecule upstream of the Imd pathway, upon exposure to *E. coli*^63^. Similarly, upon *E. coli* challenge, expression of *TmTene3*, *TmDef1*, and the AMPs listed above, was downregulated in the gut of *TmRelish*-depleted larvae. Global expression analysis of the gut epithelium of *Drosophila* following oral infection with gram-negative bacteria revealed that the Imd pathway, and not the Toll pathway, is involved in eliciting a robust immune response^64^. Several AMP genes were found to be expressed at high levels in the fat body of the *TmRelish* non-knockdown groups; however, *TmRelish* knockdown downregulated the expression of *TmTene2*, *TmTene3*, *TmTene4*, *TmDef1*, *TmDef2*, *TmCec2*, *TmCole1*, *TmCole2*, *TmAtta1a*, and *TmAtta1b*. Eliminating *TmRelish* had profound consequences on Imd pathway activation and AMP expression in both the fat body and the gut, in response to *E. coli*, suggesting that these immunocompetent tissues are a vital part of the immune response in terms of AMP production following *E. coli* infection. When evaluating mortality rate, upon *S. aureus* infection, the role of *TmRelish* in different tissues is unexpected. However, new evidence supporting the role of the Imd pathway against *S. aureus* in the *Drosophila* gut suggests involvement of the Imd pathway in gram-positive clearance. In the present study, *S. aureus* infection induced *TmTene1*, *TmTene4*, *TmCole1*, *TmCole2*, *TmAtta1a*, and *TmAtta1b* in the fat body, hemocyte, gut, and MT of ds*EGFP* controls, while all six AMP genes were moderately downregulated in *TmRelish* knockdown larvae. Interestingly, eleven of the fourteen AMP genes were negatively regulated in the gut and hemocytes, but not the fat body, by *TmRelish* knockdown upon *C. albicans* challenge, suggesting the crosstalk between *Tm*DorX2-Toll pathway in regulating AMP expression^39^. In addition to the gut, the fat body, hemocytes, and other epithelial tissues such as MTs (nephridia or kidney analogues) also play important roles in immune defense by producing AMPs^65,66^. A recent transcriptional analysis of *Zophobas morio* (Coleoptera: Tenebrionidae) showed that the fat body and MTs are versatile tissues and share important functions, such as immunity, detoxification, nitrogen metabolism, and eye pigmentation. Preliminary studies using *Drosophila* MTs suggested that Imd component genes lead to the induction of AMPs in response to microbial insults. Furthermore, immune response and AMP expression in the MTs of *D. melanogaster* have been associated with developmental regulation^67^. In the present study, only five AMP genes were downregulated in the *TmRelish* knockdown larvae infected with *E. coli* and *S. aureus*.

## Conclusions

We identified a *Relish* homologue, which was expressed in all immune tissues, with the highest expression level being observed in the gut, in the coleopteran beetle, *T. molitor*. *TmRelish* expression increased during the early hours of *E. coli* infection in the hemocytes, gut, fat body, and MTs, with the highest level of expression seen in the gut tissue of the larvae. Loss of function studies, using RNAi directed against *TmRelish*, showed significant reduction in survival of the *E. coli*-, *S. aureus-*, and *C. albicans-*challenged larvae. Higher mortality was observed in the *TmRelish* knockdown and *E. coli-* infected groups than in the *S. aureus-* and *C. albicans-*infected groups, suggesting that the target gene is involved in defense against gram-negative bacteria by inducing the expression of nine AMP genes in both the fat body and the gut. Additionally, ten AMP genes were found to be downregulated in the larval fat body of *T. molitor*, in the *Tm*Relish RNAi-treated groups, in response to *S. aureus*, indicating that *TmRelish* plays an essential role in antibacterial immune response of by larval fat body and gut, in response to *E. coli* and *S. aureus* infections.

## Supplementary information

Supplementary Figure 1.
