# Author Correction: *Tm*Relish is required for regulating the antimicrobial responses to *Escherichia coli* and *Staphylococcus aureus* in *Tenebrio molitor*

**DOI:** 10.1038/s41598-020-63872-1

**Published:** 2020-04-21

**Authors:** Maryam Keshavarz, Yong Hun Jo, Bharat Bhusan Patnaik, Ki Beom Park, Hye Jin Ko, Chang Eun Kim, Tariku Tesfaye Edosa, Yong Seok Lee, Yeon Soo Han

**Affiliations:** 1 Department of Applied Biology, Institute of Environmentally Friendly Agriculture (IEFA), College of Agriculture and Life Sciences, Chonnam National University, Gwangju, 61186 Republic of Korea; 2 Department of Biotechnology, Trident Academy of Technology (TAT), F2-A, Chandaka Industrial Estate, Chandrasekharpur, Bhubaneswar, Odisha 751024 India; 3 School of Biotechnology and Life Sciences, College of Natural Sciences, Soonchunhyang University, 22 Soonchunhyangro, Shinchang-myeon, Asan, Chungchungnam-do 31538 South Korea

Correction to: *Scientific Reports* 10.1038/s41598-020-61157-1, published online 06 March 2020

The Acknowledgements section in this Article was omitted. The Acknowledgements section should read:

“This research was supported by Basic Science Research Program through the National Research Foundation of Korea (NRF) funded by the Ministry of Science, ICT, and Future Planning (Grant No. NRF-2019R1I1A3A01057848).”

In addition to this, Figure 5 is a duplication of Figure 4. The correct Figure 5 appears below as Figure 1.

**Figure 1:**
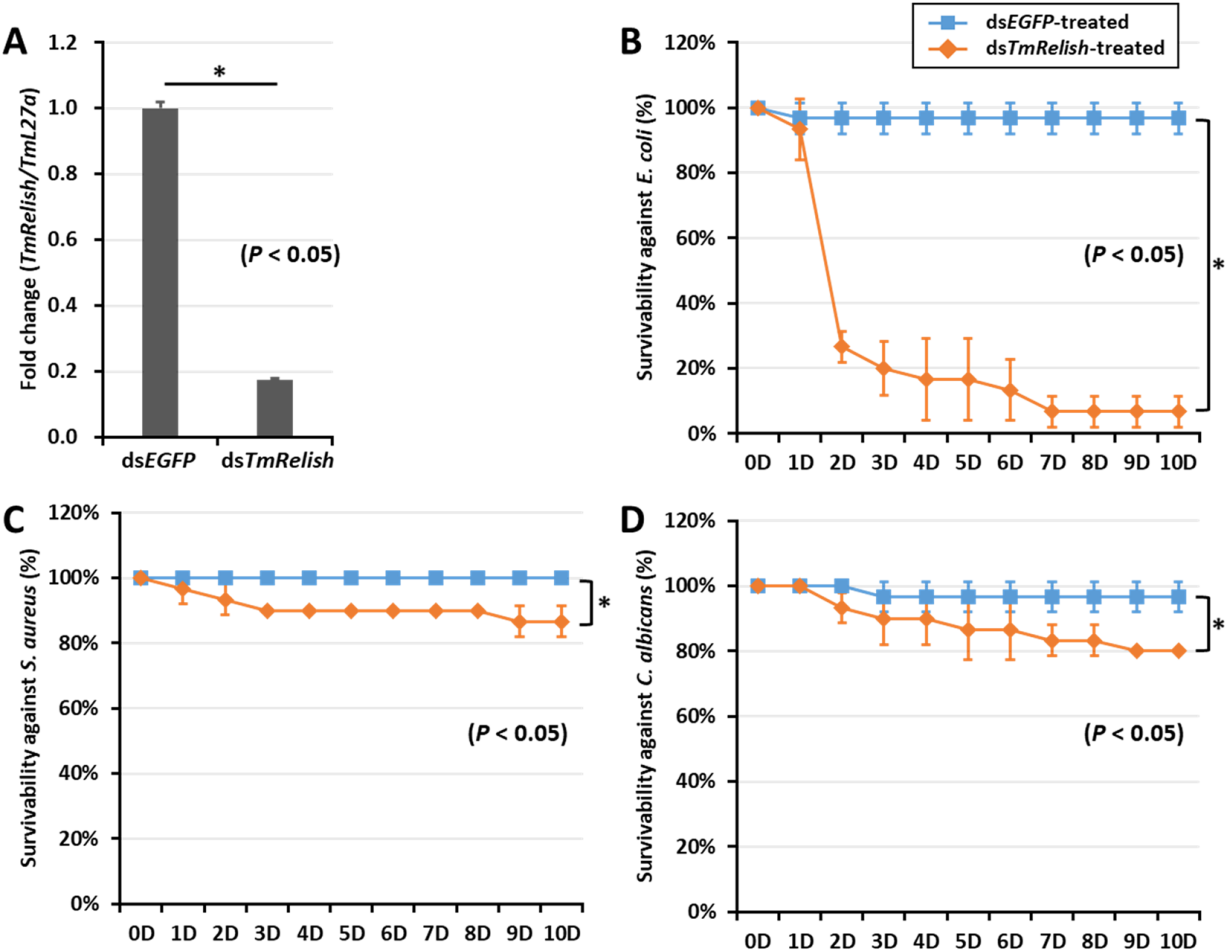
.

