# Supplementary material for: TmRelish is required for regulating the antimicrobial responses to Escherichia coli and Staphylococcus aureus in Tenebrio molitor
Source: Sci Rep. 2020 Mar 6;10:4258. doi: 10.1038/s41598-020-61157-1 (PMC7060202; doi:10.1038/s41598-020-61157-1)
Supplement: Supplementary file 1 — Supplementary Figure 1. [file 41598_2020_61157_MOESM1_ESM.docx]

Supplementary Material

***Tm*Relish is required for regulating the antimicrobial responses to *Escherichia coli* and *Staphylococcus aureus* in *Tenebrio molitor***

Maryam Keshavarz^1#^, Yong Hun Jo^1#^, Bharat Bhusan Patnaik^1,2^, Ki Beom Park^1^, Hye Jin Ko^1^, Chang Eun Kim^1^, Tariku Tesfaye Edosa^1^, Yong Seok Lee^3^, and Yeon Soo Han^1*^

^#^ These authors contributed equally to this work

*** Correspondence:**
Yeon Soo Han
[hanys@jnu.ac.kr](mailto:hanys@jnu.ac.kr)

# Supplementary Figure


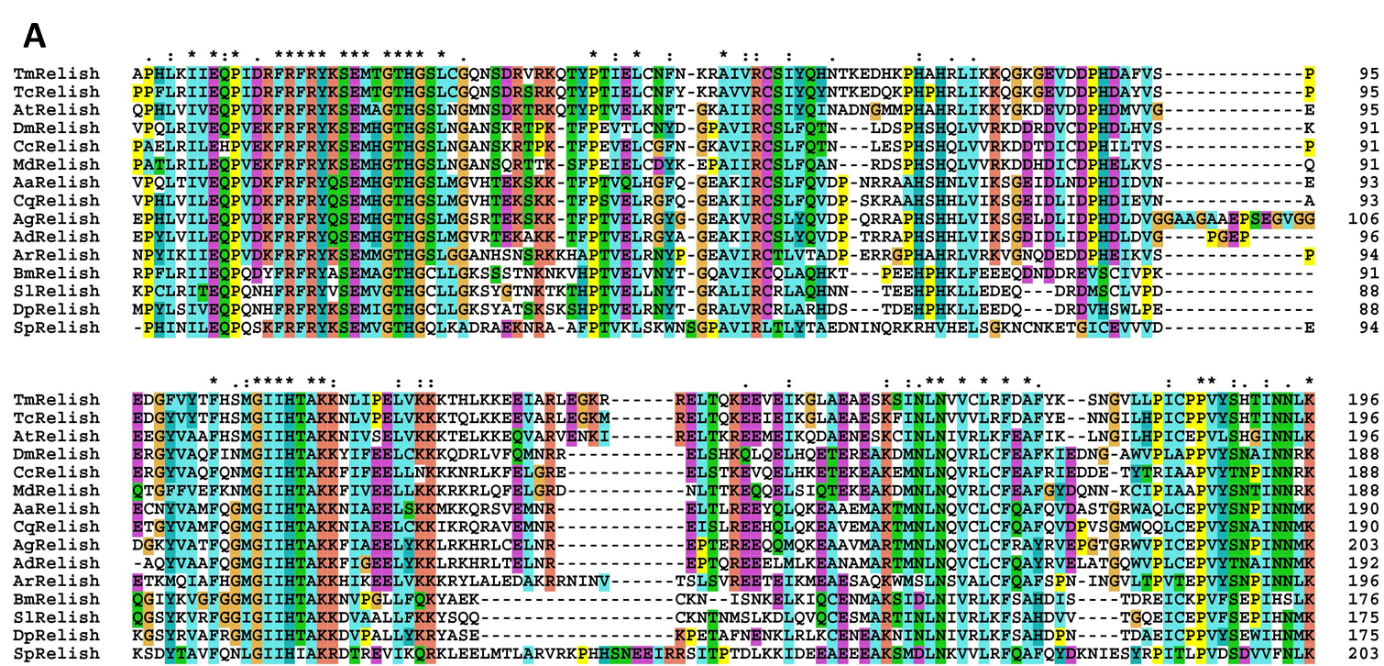


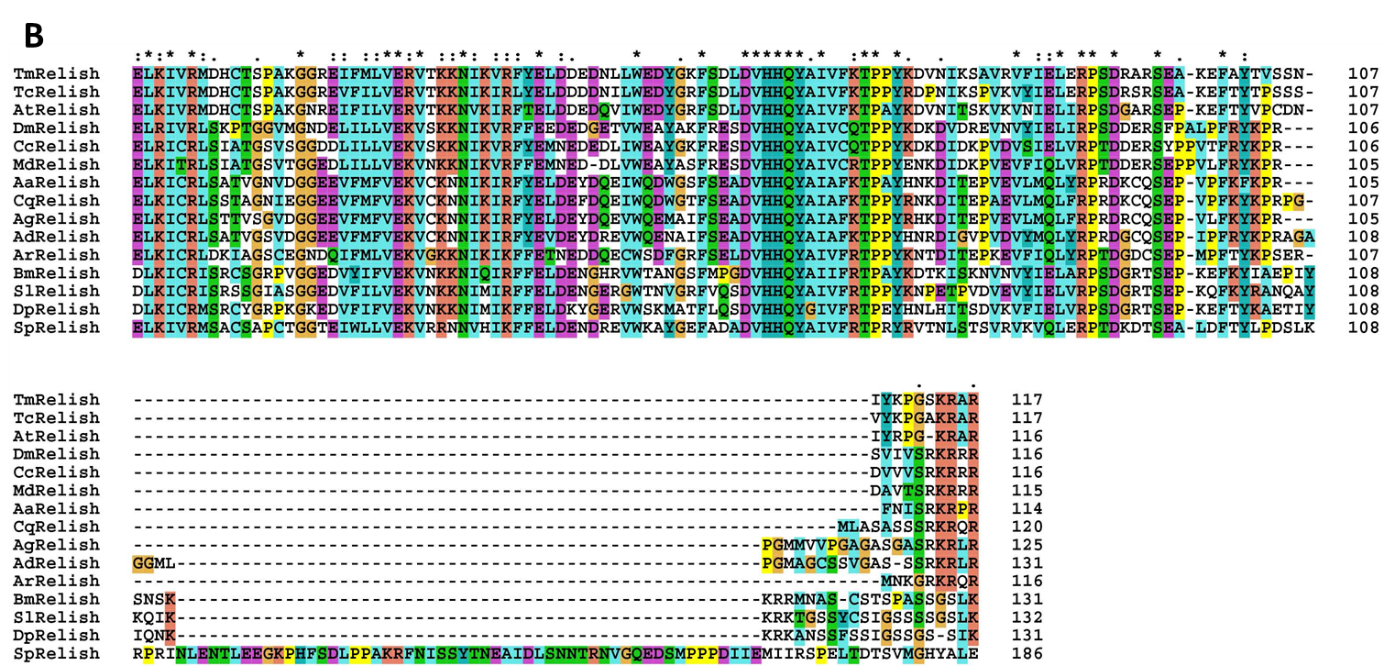


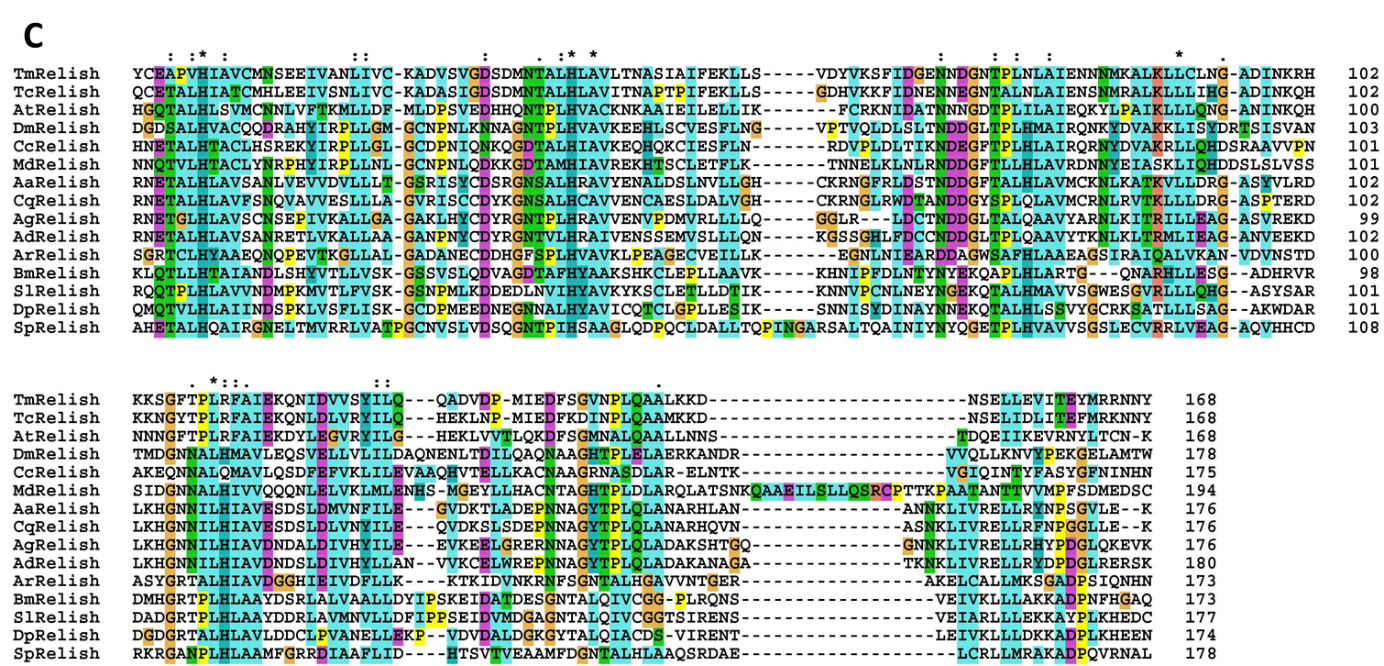


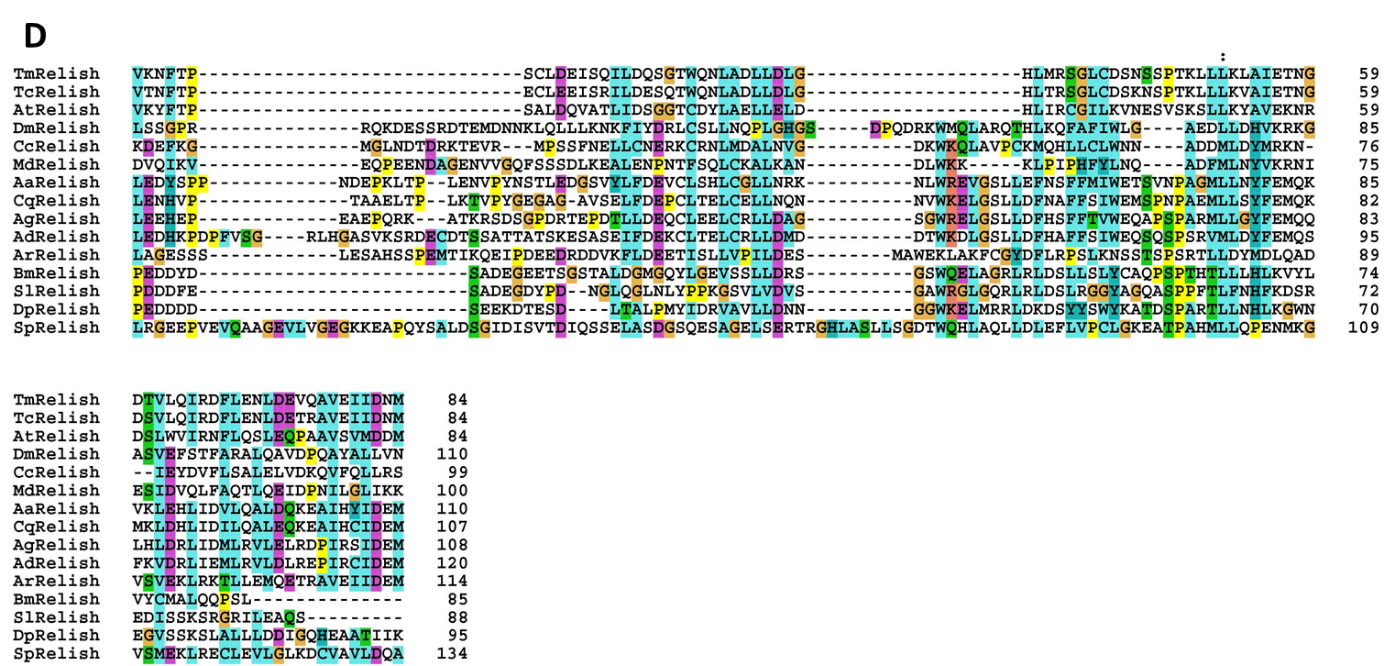


**Supplementary Figure 1.** Multiple alignments of the conserved *TmRelish* domains and the conserved Relish domain sequences of other insects obtained by clustalX2.1. ‘*’ denotes conserved amino acids; ‘:’ denotes semi-conserved residues; ‘.’ denotes less conserved residues; and ‘- ‘denotes internal or terminal gaps. The aligned protein sequences include *Tm*Relish (*T. molitor* Relish), *Bm*Relish (*Bombyx mori* Relish1, BAF74125.1), *Dm*Relish (*Drosophila melanogaster* Relish isoform D; NP_996187.1), *Aa*Relish (*Aedes aegypti* Relish isoform R6; AAM97895.1), *Ag*Relish; (*Anopheles gambiae* Relish1; AAQ57599.1), *Dp*Relish (*Danaus plexippus* Relish; OWR44464.1), *Cq*Relish (*Culex quinquefasciatus* nuclear factor NF-kappa-B p105 subunit; EDS36814.1), *Sl*Relish (*Spodoptera litura* Relish 1b; AIA24469.1), *Ad*Relish (*Anopheles darlingi* Rel 2 protein Relish; ETN65501.1), *At*Relish (*Aethina tumida* PREDICTED: nuclear factor NF-kappa-B p110 subunit, XP_019866620.1), *Cc*Relish (*Ceratitis capitata* Nuclear factor NF-kappa-B p110 subunit; JAC02247.1), *Tc*Relish (*Tribolium castaneum* Relish; EEZ97717.1), *Athalia rosae* nuclear factor NF-kappa-B p110 subunit isoform X1; *Ar*Relish (*Athalia rosae* nuclear factor NF-kappa-B p110 subunit isoform X1; XP_020709608.1), and *Md*Relish (*Musca domestica* PREDICTED: nuclear factor NF-kappa-B p110 subunit; XP_005178672.1). *Sp*Relish *(Scylla paramamosain* Relish; AZK36045.1) was used as an outgroup. The conserved Rel homology domain (RHD) (**A**), Ig-like/plexins/transcription factor (IPT) domain (**B**), Ankyrin repeats (1-5) (**C**), and death domain (DD) (**D**) of *TmRelish*.
